# MaxBin: an automated binning method to recover individual genomes from metagenomes using an expectation-maximization algorithm

**DOI:** 10.1186/2049-2618-2-26

**Published:** 2014-08-01

**Authors:** Yu-Wei Wu, Yung-Hsu Tang, Susannah G Tringe, Blake A Simmons, Steven W Singer

**Affiliations:** 1 Joint BioEnergy Institute, Emeryville, CA 94608, USA; 2 Physical Biosciences Division, Lawrence Berkeley National Laboratory, Berkeley, CA 94720, USA; 3 City College of San Francisco, San Francisco, CA 94112, USA; 4 Joint Genome Institute, Walnut Creek, CA 94598, USA; 5 Genomics Division, Lawrence Berkeley National Laboratory, Berkeley, CA 94720, USA; 6 Biological and Materials Sciences Center, Sandia National Laboratories, Livermore, CA 94551, USA; 7 Earth Sciences Division, Lawrence Berkeley National Laboratory, Berkeley, CA 94720, USA

**Keywords:** Binning, Metagenomics, Expectation-maximization algorithm

## Abstract

**Background:**

Recovering individual genomes from metagenomic datasets allows access to uncultivated microbial populations that may have important roles in natural and engineered ecosystems. Understanding the roles of these uncultivated populations has broad application in ecology, evolution, biotechnology and medicine. Accurate binning of assembled metagenomic sequences is an essential step in recovering the genomes and understanding microbial functions.

**Results:**

We have developed a binning algorithm, MaxBin, which automates the binning of assembled metagenomic scaffolds using an expectation-maximization algorithm after the assembly of metagenomic sequencing reads. Binning of simulated metagenomic datasets demonstrated that MaxBin had high levels of accuracy in binning microbial genomes. MaxBin was used to recover genomes from metagenomic data obtained through the Human Microbiome Project, which demonstrated its ability to recover genomes from real metagenomic datasets with variable sequencing coverages. Application of MaxBin to metagenomes obtained from microbial consortia adapted to grow on cellulose allowed genomic analysis of new, uncultivated, cellulolytic bacterial populations, including an abundant myxobacterial population distantly related to *Sorangium cellulosum* that possessed a much smaller genome (5 MB versus 13 to 14 MB) but has a more extensive set of genes for biomass deconstruction. For the cellulolytic consortia, the MaxBin results were compared to binning using emergent self-organizing maps (ESOMs) and differential coverage binning, demonstrating that it performed comparably to these methods but had distinct advantages in automation, resolution of related genomes and sensitivity.

**Conclusions:**

The automatic binning software that we developed successfully classifies assembled sequences in metagenomic datasets into recovered individual genomes. The isolation of dozens of species in cellulolytic microbial consortia, including a novel species of myxobacteria that has the smallest genome among all sequenced aerobic myxobacteria, was easily achieved using the binning software. This work demonstrates that the processes required for recovering genomes from assembled metagenomic datasets can be readily automated, an important advance in understanding the metabolic potential of microbes in natural environments. MaxBin is available at https://sourceforge.net/projects/maxbin/.

## Background

The development of high-throughput genomic sequencing technologies has enabled the recovery of genomes directly from microbial communities in natural and engineered environments [1]. Genomes have been recovered from microbial communities found in acid mine drainage [2,3], permafrost [4], cow rumen [5], surface ocean water [6], sludge bioreactors [7], acetate-amended aquifers [8], and infant fecal samples [9]. A key step in genome recovery from metagenomic sequence data is the classification of sequences assembled from metagenomic reads into discrete units, referred to as bins. These bins represent composite genomes of individual populations that comprise the microbial community. A number of approaches have been developed to bin assembled sequences from metagenomic data [2,4-9]. Among these techniques, one of the most widely used is emergent self-organizing maps (ESOMs), which have been used to bin assembled sequences by tetranucleotide frequencies [2] and read coverage levels (time series binning) [9]. ESOMs calculated based on tetranucleotide frequencies can be applied to individual metagenomic datasets; however, time series ESOMs require multiple datasets for accurate binning. A related approach to time series ESOM binning is differential coverage binning, which uses plots of differential read coverages of assembled sequences to distinguish individual genomic bins. In both methods, individual bins are tested for completeness (is it a complete genome?) and distinctiveness (does the bin only contain one genome?) using single-copy marker genes.

For both ESOM and differential coverage binning approaches, individual bins are chosen manually from a graphical output. Existing automated binning algorithms, such as AbundanceBin [10] or MetaCluster [11,12], are designed to bin sequencing reads instead of assembled metagenomic scaffolds. Here, we describe the development of a novel binning method, MaxBin, which automates binning of assembled metagenomic scaffolds using an expectation-maximization algorithm. In this approach, tetranucleotide frequencies and scaffold coverages are combined to organize metagenomic sequences into individual bins, which are predicted from initial identification of marker genes in assembled sequences. The performance of MaxBin was evaluated on simulated metagenomic datasets, individual datasets obtained in the Human Microbiome Project and replicates of cellulolytic consortia enriched from compost. Genomic analysis of the members of the cellulolytic consortia revealed multiple uncultivated cellulolytic populations, including a recovered myxobacterial genome distantly related to *Sorangium cellulosum* that is substantially smaller than most known myxobacterial genomes.

## Methods

**Figure 1:**
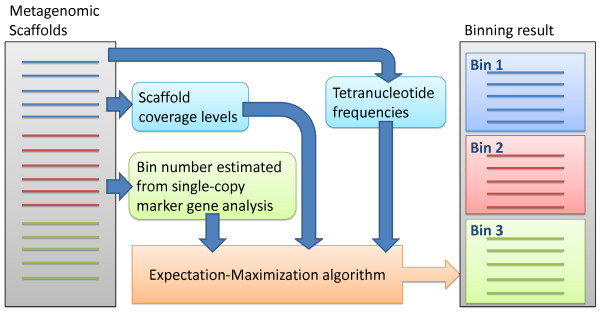
**The general workflow of MaxBin.** Tetranucleotude frequencies, scaffold coverage levels, and single-copy marker genes are collected from metagenomic scaffolds. The collected information is computed by an expectation-maximization algorithm to bin sequences.

A flow diagram for the operation of the MaxBin algorithm is shown in Figure 1. Before applying the MaxBin binning algorithm on any dataset, the sequencing reads need to be assembled into contigs or scaffolds, which are contigs linked by Ns based on paired-end information. Below, we will use scaffolds to describe these assembled sequences. MaxBin is capable of binning either contigs or scaffolds; see the expectation-maximization algorithm section below for details. The MaxBin algorithm utilizes two different genomic features: tetranucleotide frequencies and scaffold coverage levels to populate the genomic bins using single-copy maker genes and an expectation-maximization algorithm. After classifying the scaffolds into different bins, MaxBin produces an optimal set of genomic bins and reports the estimated genomic features, including: genome sizes, GC content, genome completeness, and genome coverage levels, and provides this information in tabular form.

### Probability estimations of genomic features

Genomic signatures have been shown to display a species-specific pattern [13-16] and have been applied to bin sequences from metagenomic datasets. The most widely used genomic signature is tetranucleotide frequencies, which has been used in a number of metagenomic studies [4-8]. To distinguish whether two sequences are sampled from the same species based on their tetranucleotide frequencies, we downloaded 3,181 bacterial and archaeal genomes from the IMG website (http://img.jgi.doe.gov/), simulated genomic sequence fragments, and calculated Euclidean distance between the extracted tetranucleotide frequencies of the two sequences. The lengths of the simulated sequences were randomly chosen between 1,000 bps to 1,000,000 bps. The simulation was performed one million times for intra-genome (sequences sampled from the same genome) and one million times for inter-genome (sequences sampled from different genomes) comparisons. The histogram of intra-genome and inter-genome simulations, as shown in Additional file 1: Figure S1(A) and S1(B), revealed a large difference, in which intra-genome distances were grouped below 0.2 while inter-genome distances were more evenly distributed between 0.02 and 0.1. We estimated both the mean and variance values separately for both intra-genome and inter-genome Euclidean distances. The mean and standard deviation values for intra- and inter-genome distances were 0.015, 0.010 and 0.068, 0.034, respectively. We must note that both distributions of intra- and inter-genome distances were rejected by the Shapiro-Wilk test to be normally distributed (W value is 0.81 and 0.93 for intra- and inter-genome distances, respectively); however, because the difference between the histograms of intra- and inter-genome distances is very clear, we still applied their mean and standard deviation values to test whether the measured Euclidean distances are between sequences sampled from the same genome or between different genomes. We discuss this circumstance and potential improvement in the Discussion section.

After identifying the mean and standard deviation values between the distributions of Euclidean distance, as shown in Additional file 1: Figure S1(C), we define the distance function between two sequences as

$$\mathrm{dist}\left(S_{1},S_{2}\right)=\mathrm{dis}t_{\mathrm{Euc}}\left(\mathrm{tetra}\left(S_{1}\right),\mathrm{tetra}\left(S_{2}\right)\right)$$

where *tetra (S)* indicates the tetranucleotide frequency of sequence *S*, and $\mathrm{dis}t_{\mathrm{Euc}}\left(\right)$ function is used to calculate the distances between the two sequences, *S*_1_ and *S*_2_, using Euclidean distance function. The probability function that two sequences were sampled from the same species is defined as:

$$P_{\mathrm{dist}}\left(S_{1}\in G\left(S_{2}\right)\right)=\frac{N_{\mathrm{intra}}\left(\mathrm{dist}\left(S_{1},S_{2}\right)\;|\mu_{\mathrm{intra}},\sigma_{\mathrm{intra}}^{2}\right)}{N_{\mathrm{intra}}\left(\mathrm{dist}\left(S_{1},S_{2}\right)\;|\;\mu_{\mathrm{intra}},\sigma_{\mathrm{intra}}^{2}\right)+N_{\mathrm{inter}}\left(\mathrm{dist}\left(S_{1},S_{2}\right)\;|\;\mu_{\mathrm{inter}},\sigma_{\mathrm{inter}}^{2}\right)}$$

in which $G\left(S_{2}\right)$ represents the genome that sequence *S*_2_ belongs to, $\mathrm{dist}\left(S_{1},S_{2}\right)$ is the distance between the extracted tetranucleotide frequencies of *S*_1_ and *S*_2_ using the Euclidean distance function, *N*_intra_ and *N*_inter_ are the Gaussian distributions with estimated intra- and inter-genome mean distance values (*μ*_*intra*_ and *μ*_*inter*_) and distance variance values $\left(\sigma_{\mathrm{intra}}^{2}\;\mathrm{and}\;\sigma_{\mathrm{inter}}^{2}\right)$, respectively (note that *P*_*dist*_(*S*_1_ ∈ *G*(*S*_2_)) = *P*_*dist*_(*S*_2_ ∈ *G*(*S*_1_)) since *dist*(*S*_1_, *S*_2_) = *dist*(*S*_2_, *S*_1_). The distribution *P*_*dist*_ for Euclidean distance is shown in Additional file 1: Figure S1(D). One can easily observe from the figure that the lower the distance, the more probable two sequences are sampled from the same genome.

The scaffold coverage levels are also considered, as coverage levels also carry important information and have been applied to bin metagenomic data [7,9]. Shotgun sequencing has been demonstrated to follow the Lander-Waterman model, which calculates the coverage of the sequenced nucleotides using a Poisson distribution [17] and has been applied in the binning of metagenomic reads [10,18]. The probability function that two sequences are sampled from the same genome given their coverages is modeled as:

$$P_{\mathrm{cov}}\left(S_{1}\in G\left(S_{2}\right)\right)=\mathrm{Poission}(\mathrm{cov}\left(S_{1}\right)|\mathrm{cov}\left(S_{2}\right)$$

where *cov*(*S*) indicates the coverage of sequence *S*, and *Poisson* (cov(*S*_1_) | cov(*S*_2_)) is the Poisson probability density function given mean value λ = *cov* (*S*_*2*_).

### Expectation-maximization algorithm

MaxBin utilizes tetranucleotide frequencies and scaffold coverage levels to estimate the probability that a scaffold belongs to a bin using an expectation-maximization (EM) algorithm. The algorithm consists of five steps as follows:

1. Estimate the tetranucleotide frequencies and coverage levels for all scaffolds. Tetranucleotide frequencies are calculated by scanning the number of all possible tetramers (that is, four consecutive nucleotides) using one bp sliding window on both forward and reverse-complement strands of any scaffold. Tetramers with non-nucleotide symbols (that is, not A, T, C, or G) are discarded. Since DNA fragments can be obtained from either strand of the genomes, the frequency of one tetramer and its reverse-complement is combined, resulting in a total of 136 possible tetramers.

2. Initialize the total number of genomes *N*, their inherent tetranucleotide frequencies *tetra*_*i*_ and coverage levels *λ*_*i*_ for *i* = 1,2…, *N*.

3. Calculate the probability that any sequence *S*_*j*_ (*j* = 1, 2…, *W*; *W* is the total number of scaffolds) coming from the *i*_*th*_ genome *G*_*i*_ as

$$P(S_{j}\in G_{i})=\frac{P_{\mathrm{dist}}\left(S_{j}\in G_{i}\right)\cdot P_{\mathrm{cov}}\left(S_{j}\in G_{i}\right)}{\sum_{i=1}^{N}P_{\mathrm{dist}}\left(S_{j}\in G_{i}\right)\cdot P_{\mathrm{cov}}\left(S_{j}\in G_{i}\right)}$$

4. Calculate the new values for each *tetra*_*i*_ and *λ*_*i*_ as

$$\begin{array}{l} \mathrm{tetr}a_{i}=\frac{\sum_{j=1}^{W}P\left(S_{j}\in G_{i}\right)\cdot \mathrm{tetra}\left(S_{j}\right)\cdot \mathrm{lenght}\left(S_{j}\right)}{\sum_{j=1}^{W}P\left(S_{j}\in G_{i}\right)\cdot \mathrm{lenght}\left(S_{j}\right)}\\ \lambda_{i}=\frac{\sum_{j=1}^{W}P\left(S_{j}\in G_{i}\right)\cdot \mathrm{cov}\left(S_{j}\right)\cdot \mathrm{lenght}\left(S_{j}\right)}{\sum_{j=1}^{W}P\left(S_{j}\in G_{i}\right)\cdot \mathrm{lenght}\left(S_{j}\right)} \end{array}$$

5. Iterate step 3 and 4 until the parameters converge or the number of runs exceeds a pre-defined maximum number of runs. The maximum number of runs is set to 50.

The EM algorithm calculates the probability that a given scaffold belongs to any genome at the same time. The maximum number of iterations of the EM algorithm was determined by running MaxBin on simulated datasets with different maximum iteration numbers and measuring the performances of the binning results by precision and sensitivity (see below). As shown in (Additional file 1: Figure S2), the precision and sensitivity of the two simulated datasets were very stable for all maximum iteration number settings. We have set the maximum number to perform EM algorithm to 50 in case some larger metagenomes need more iterations to achieve reasonable binning results.

After the EM algorithm is finished, the scaffolds are assigned to the bin with the highest probability as long as the probability values surpass the minimum probability threshold, which is set to 80%. Sequences that do not meet the threshold are discarded as ‘unclassified.’

Before applying the EM algorithm on any metagenomic datasets, sequences shorter than minimum length threshold are removed since these sequences are likely to produce skewed tetranucleotide frequencies, which confuse the binning algorithm and erroneously classify shorter sequences into wrong bins. To find the best minimum length cutoff threshold, we performed binning using different length cutoff settings on simulated datasets. The result demonstrated that MaxBin achieves the best sensitivity and comparable precision with 1,000 bps cutoff setting [see Additional file 1: Figure S3]. Therefore, we set the minimum length threshold to 1,000 bps to achieve best performances.

### Initialization of the algorithm

A common practice for an expectation-maximization algorithm is to randomize all parameters. This initial condition permits the possibility of converging parameters into local maxima. At the same time, the number of bins, which is one of the most crucial parameters, is usually unknown. We employed the single-copy marker genes to estimate the number of bins and initialize all required parameters. Genes from the scaffolds were predicted using FragGeneScan [19], and HMMER3 [20] was used (with --cut_tc option) to scan the predicted genes for 107 single-copy marker genes that are conserved in 95% of all sequenced bacteria, which has been used to determine the genome completeness of bins [7,21]. After filtering out all mapped genes that do not meet the coverage threshold, which is 40%, the median number of scaffolds containing each of the marker genes is identified, considering that some marker genes may be fragmented into several pieces that may distort the estimation of the number of bins. The shortest marker gene that corresponds to the median number of bins is selected, and the tetranucleotide frequencies and read coverages of the scaffolds harboring this shortest marker gene are extracted to generate the initialization parameters for the algorithm. The reason we use the shortest marker gene for initialization is that shorter genes are less likely to be split between two scaffolds.

### Recursive classification of bins

Despite careful selection of initialization conditions, the EM algorithm sometimes may still group scaffolds from several composite genomes into one bin. To alleviate this problem, all bins are recursively checked for the median number of marker genes. If the median number of marker genes of any bin is at least 2, the bin will be treated as a dataset waiting to be binned, and the whole EM algorithm will be applied to split the bin. All bins (including those created by reapplying EM algorithm on bins) will be checked for the number of marker genes until no bins can be split further.

### Estimation of genome completeness

After the EM process, MaxBin scans the 107 marker genes in all bins for genome completeness, which is measured as the fraction of unique marker genes versus all marker genes. Not all bacteria are equipped with all 107 marker genes, such as the reconstructed genomes from the uncultivated TM7 lineage, in which representatives of this candidate phylum have only 100 out of all 107 marker genes [7]. However, the metric of 107 marker genes is maintained as the standard in the absence of specific phylum level counts of conserved marker genes.

### Simulation of the test metagenomes

Simulated metagenomes with 10 species and 100 species were generated by MetaSim [22] and assembled by Velvet assembler 1.2.07 [23] with K = 55 and coverage cutoff set to 1. The detailed genome simulation settings can be found in Additional file 1: Table S1 and S2. For 10-species metagenomes, we sampled 5 million and 20 million paired-end reads referred to as the 20X and 80X datasets, respectively. For 100-species metagenomes, the settings used by [24] were mimicked, and 100 million paired-end reads were sampled to create three datasets: simLC+, simMC+, and simHC+. The 80-bps error model was downloaded from the MetaSim website (http://ab.inf.uni-tuebingen.de/software/metasim/errormodel-80bp.mconf/view) and used in simulating all metagenomes.

### Performance evaluation of the binning results of simulated datasets

We adopted precision and sensitivity from [12] to evaluate the binning performances of the simulated datasets. Assume there are N genomes in the dataset and MaxBin yielded M bins. The overall precision and sensitivity is given as

$$\begin{array}{l} \mathrm{Precision}=\frac{\sum_{i=1}^{M}\max_{j}R_{\mathrm{ij}}}{\sum_{i=1}^{M}\sum_{j=1}^{N}\max_{j}R_{\mathrm{ij}}}\\ \mathrm{Sensitivity}=\frac{\sum_{j=1}^{N}\max_{i}R_{\mathrm{ij}}}{\sum_{i=1}^{M}\sum_{j=1}^{N}\max_{j}R_{\mathrm{ij}}+\mathrm{unclassified}\;\mathrm{sequences}} \end{array}$$

in which *R*_*ij*_ indicate the number of sequences (in terms of base pairs) that belong to genome *j* appears in bin *i*. If *M* > *N*, the majority of sequences in each bin likely belong to a single genome and the precision will be high; however, the sensitivity will be low since some genomes are represented by more than one bin. On the other hand if *M* < *N*, the sensitivity will tend to be high while precision is likely to be low. We also note that scaffolds lower than 1,000 bps, which is our minimum length cutoff for MaxBin algorithm, are not included in the unclassified sequences since these sequences cannot be binned and will be discarded in the first step of the binning algorithm.

Besides precision and sensitivity, we also evaluated the amount of correctly binned and mis-assigned sequences for individual species in the simulated datasets. We evaluated the number of sequences of each species that appear in each bin, and assigned the bins to the species with the highest amount of sequences in the bins. If two or more bins are assigned to the same species, only the bin with the greatest amount of sequences belonging to that species is kept, and only sequences belonging to that species in that bin are regarded as correctly binned; all sequences appear in other bins and sequences that do not belong to the assigned species in any bin are regarded as mis-assigned. Unclassified sequences are defined as sequences that pass the minimum length threshold but are not classified into any bins. Sequences lower than the minimum length threshold are not considered here since these sequences are not treated as part of the binning result.

### Test environment

MaxBin was tested on a Linux operation system with 128G memory space and 16 AMD Opteron™ CPU cores at 2.2 GHz. With the exception of HMMER3, which MaxBin utilized to extract marker gene information, MaxBin itself and other affiliated software are not multi-threaded. The running time of MaxBin for all simulated and real datasets is reported in Additional file 1: Table S8. Note that MaxBin did not consume more than 1GB of memory space for all our test datasets except when we map reads against scaffolds to obtain scaffold coverage information, suggesting that the MaxBin algorithm could be executed on a personal computer.

### Sequencing the enriched cellulolytic compost samples

Green waste compost samples were obtained from the City of Berkeley, CA, on 30 June 2012. Replicate 50 mL cultures (37A and 37B) containing MES-buffered M9TE minimal media [25] were established with microcrystalline cellulose (500 mg, 1% v/v) (Sigma-Aldrich) as the carbon substrate. These cultures were incubated on rotary shakers for two weeks at 37°C and 200 rpm. Five milliliters of this culture (10%) was transferred to a second replicate set of microcrystalline cellulose-containing cultures and incubated for an additional two weeks. After the second passage, DNA was extracted from culture biomass using previously described methods [26]. DNA fragments for Illumina sequencing were created using the Joint Genome Institute standard library generation protocols for Illumina HiSeq 2000 platforms. Illumina sequencing was performed on a HiSeq 2000 system. The DNA fragments were assembled using the Joint Genome Institute assembly pipeline. Raw reads were trimmed using a minimum quality cutoff of Q10. Trimmed, paired-end Illumina reads were assembled using SOAPdenovo v1.05 (http://soap.genomics.org.cn/soapdenovo.html) with default settings (-d 1 and -R) at different Kmer sizes (85, 89, 93, 97, 101 and 105 respectively). Contigs generated by each assembly (a total of six contig sets from the six Kmer sizes), were merged using in-house Perl scripts as following. Contigs were first de-replicated and sorted into two pools based on length. Contigs <1,800 bps were assembled using Newbler (Life Technologies, Carlsbad, CA, USA) to generate larger contigs (-tr, -rip, -mi 98, -ml 80). All assembled contigs >1,800 bps, as well as the contigs generated from the Newbler assembly, were combined and merged using minimus2 (-D MINID = 98 -D OVERLAP = 80) (AMOS: http://sourceforge.net/projects/amos). The average fold coverage (or read depth) of each scaffold was estimated by mapping all Illumina reads back to the final assembly using BWA (version 1.2.2) [27].

### Extraction of glycoside hydrolase genes from the *Sorangium* sp. bin

Glycoside hydrolase (GH) genes of the *Sorangium* sp. binned genomes and the two *Sorangium cellulosum* strains were extracted using dbCAN [28], which was based on the protein families generated by CAZy database (http://www.cazy.org/) [29]. The extracted GH families were then grouped based on [30], in which the GH families were classified into cellulases (GH5, 6, 7, 9, 44, 45, 48), endohemicellulases (GH8, 10, 11, 12, 26, 28, 53), cell wall elongation enzymes (GH16, 17, 74, 81), de-branching enzymes (GH51, 54, 62, 67, 78), and oligosaccharide-degrading enzymes (GH1, 2, 3, 29, 35, 38, 39, 42, 43, 52). A new protein class, ‘lignin-degradation enzymes,’ was defined by including the protein families from AA1 to AA8 as suggested by [31].

### Construction of phylogenetic trees

The 16S ribosomal RNA genes were extracted and aligned using MUSCLE [32]. The alignments were refined using Gblocks [33] and loaded into MEGA5 [34] to construct the maximum-likelihood tree using default settings (Tamura-Nei model, uniform rates, and complete deletion) with 1,000 bootstraps. The marker gene trees were built using the 35 marker genes previously reported [35]. The 35 marker genes were extracted from the downloaded or binned genomes, translated to amino acid sequences, and aligned by MUSCLE separately. The alignments were then concatenated and refined using Gblocks. MEGA5 was again used to build the maximum-likelihood marker gene tree using default settings (JTT model, uniform rate, and complete deletion) with 1,000 replicates.

### Identifying clusters of orthologous groups families

Multiple alignments of all clusters of orthologous groups (COGs) were downloaded from eggNOG website (http://eggnog.embl.de/) [36] and converted to hidden Markov models using HMMER3 [20]. The functional categories of all COGs were also downloaded from eggNOG website for counting the numbers of genes for each functional category.

### Pathway mapping

Proteins extracted from *Sorangium* sp. were searched for their KEGG (Kyoto Encyclopedia of Genes and Genomes) Orthology (KO) numbers using KEGG2 KAAS web service [37]. The resulting KO numbers were inputted into the Search&Color Pathway web service (http://www.genome.jp/kegg/tool/map_pathway2.html) available on the KEGG2 website [38] to identify the associated pathways.

### Data access

The MaxBin program is available at https://sourceforge.net/projects/maxbin/. Metagenomic data, including raw sequencing reads and assembled sequences, for the enriched cellulolytic compost consortia are available at JGI IMG/M website (https://img.jgi.doe.gov/cgi-bin/m/main.cgi) under JGI taxon id 3300000869 (37A) and 3300001258 (37B). The MetaSim setting files, assembled scaffolds, and coverage files for replicating the simulation results, the binning results of HMP datasets that were mentioned in the text, and the binning results of the enriched cellulolytic compost metagenomes can be downloaded from the MaxBin download page (http://downloads.jbei.org/data/MaxBin.html).

## Results

### Testing MaxBin on simulated metagenomes

**Figure 2:**
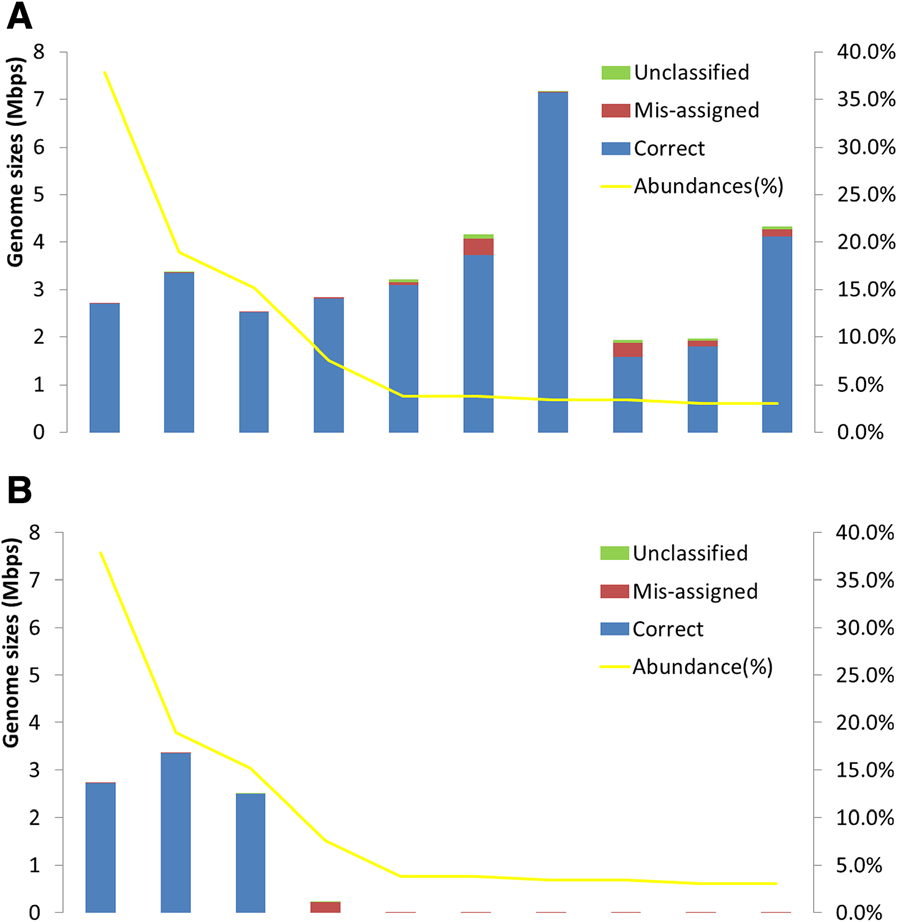
**Binning performance estimated from two 10-genome simulated datasets.** Only scaffolds longer than 1,000 bps were used. Each bar represents an individual genome; blue, red, and green parts indicate correctly assigned, mis-assigned, and unclassified scaffolds, respectively. In other words, the presence of red bars indicates that part of the genome has been incorrectly assigned to bins belonging to other species, and green bars are genome parts that are unclassified. These do not count scaffolds shorter than 1,000 bps since these scaffolds will be discarded before applying the expectation-maximization algorithm and do not reflect the performance of MaxBin. Yellow lines represent the relative abundance ratios of the corresponding species. The Y-axis at the left and right side indicate binned genome sequences in million bps and genome abundances in relative abundance ratio (%). The species names of the genomes were indicated in Additional file 1: Table S1. **(A)** 80X simulation. **(B)** 20X simulation. An entire red bar, such as the fourth bin in the 20X simulation, indicates that scaffolds of this species have been incorrectly assigned to other bins. Genomes with much shorter bars indicate that these genomes were assembled poorly and hence only a small proportion of scaffolds are longer than 1,000 bps and show up in the figures.

MaxBin has been designed as an automated metagenomic binning software, which allows binning of assembled metagenomic scaffolds after the assembly of metagenomic sequencing reads with minimal human intervention. MaxBin was initially tested by binning several simulated metagenomic datasets produced by MetaSim [22] to evaluate its effectiveness. MaxBin was applied to two simulated metagenomes containing 10 species with different overall sequencing coverage (20X versus 80X average coverage). The species used in this simulation and their relative abundance ratios, defined by the actual coverage levels divided by summed coverage levels of all genomes, were summarized in Additional file 1: Table S1. MaxBin was first interrogated for its ability to classify sequences correctly into corresponding bins. MaxBin successfully binned the 80X metagenome into 10 bins, in which the majority of sequences were correctly classified (Figure 2(A)). High abundance genomes were binned almost perfectly; most of the erroneously binned sequences occurred in low abundance genomic bins with similar coverage levels. The precision was estimated to be 96.9%, as shown in Table 1. These results demonstrated the ability of MaxBin to estimate correctly the number of bins as well as utilize tetranucleotide frequencies and scaffold coverage information to bin most of the sequences accurately.

**Table 1:** Binning performances of simulated datasets

**Datasets**		**Bin number**	**Precision**	**Sensitivity**^**a**^	**Unclassified**^**a**^
10 genomes	20X	3	97.01%	96.15%	0.002 Mbps
	80X	10	96.90%	99.34%	0.30 Mbps
100 genomes	simLC+	57	65.07%	62.83%	41.18 Mbps
	simMC+	11	74.95%	92.41%	0.68 Mbps
	simHC+	78	88.93%	73.46%	62.50 Mbps

^a^Scaffolds shorter than 1,000 bps are not included in the estimation of sensitivity and the total amount of unclassified sequences.

The metagenome with 20X average coverage was binned into three bins, each consisting of sequences from the most abundant three genomes (Figure 2(B)). Due to the lower sequencing coverages of the seven low abundance genomes, the majority of assembled scaffolds that belong to these low abundance genomes did not pass the minimum length threshold and cannot be binned by MaxBin. Therefore, only three bins were produced from the 20X metagenome, each belonging to the three genomes with highest abundances. Most of the scaffolds belonging to the seven low abundance genomes were discarded because these scaffolds did not pass the length threshold. For most binning methods, 1,000 bps is the minimum length to bin scaffolds successfully (see Discussion for details). Despite these limitations, MaxBin still binned the three most abundant genomes with precision measured at 97.01%. We note that for both 80X and 20X samples, the unclassified sequences were both lower than 1 Mbps (Table 1; sequences below minimum length threshold were not included), suggesting that MaxBin was able to classify most of the sequences while committing very few mis-assigned errors.

**Figure 3:**
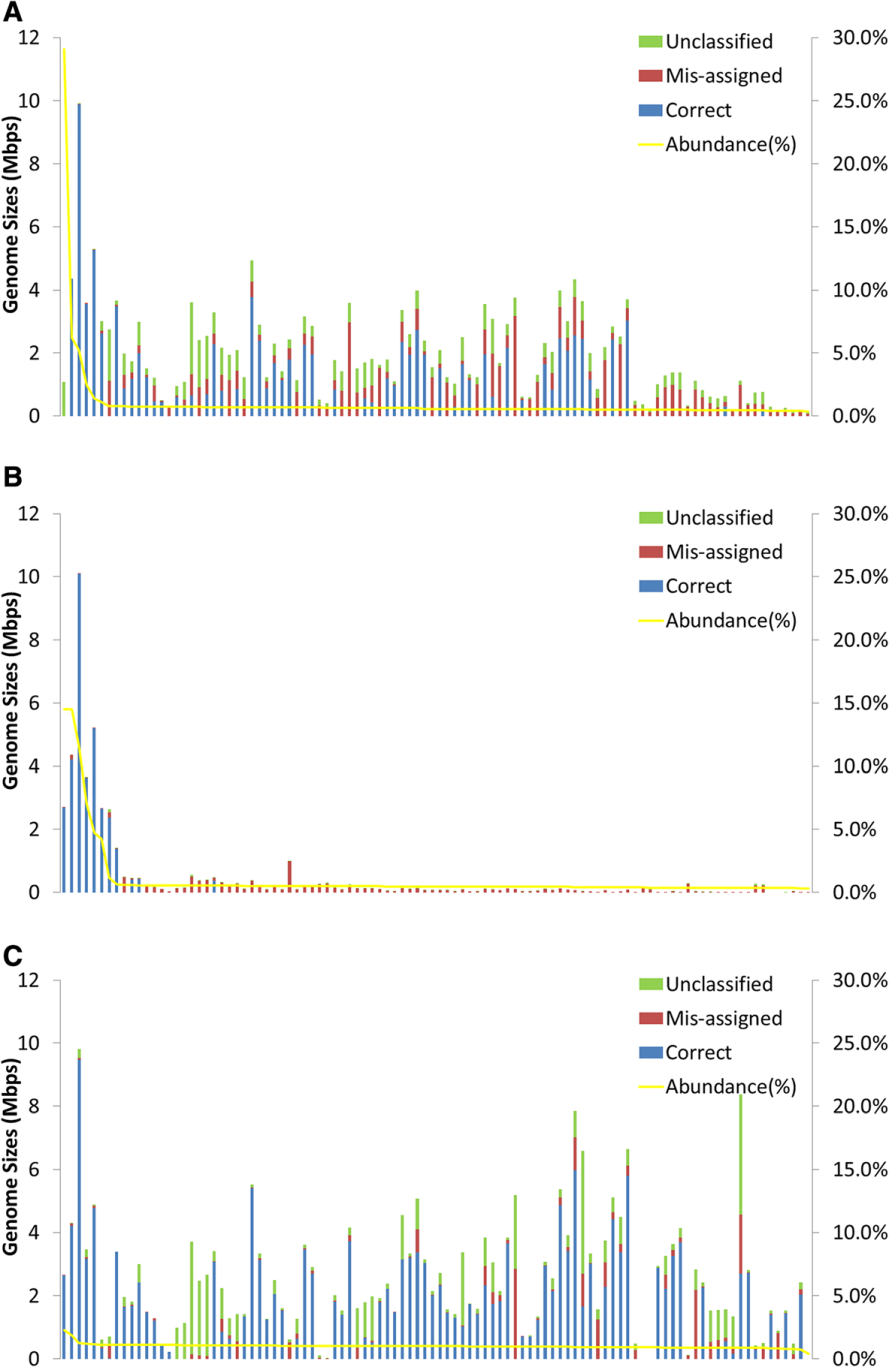
**Binning performance estimated from three 100-genome simulated datasets.** The graph settings are the same as for Figure 2. The species names of the genomes were indicated in Additional file 1: Table S2. **(A)** simLC + simulation. **(B)** simMC + simulation. **(C)** simHC + simulation.

The binning capabilities of MaxBin were further tested on more complex metagenomic datasets. Genome coverage settings of three previously published simulated metagenomic datasets were generated (simLC, simMC, and simHC) [24], and three datasets were produced with similar settings: simLC+, simMC+, and simHC + [see Additional file 1: Table S2]. MaxBin isolated 57 bins from simLC+, 11 bins from simMC+, and 78 bins from simHC+. The precision, sensitivity, and the amount of unclassified sequences are also summarized in Table 1. Overall, sequences from high abundance genomes were binned more accurately and more comprehensively than low abundance genomes as demonstrated for the simLC + and simMC + datasets (Figure 3(A)-(B)). The first few genomes with high abundance levels were binned very accurately; the majority of mis-assigned scaffolds occurred in low-abundance bins. Investigation of the assembled sequences of simLC + and simMC + demonstrated that the correctly assigned scaffolds were significantly longer than mis-assigned ones [see Additional file 1: Figure S4]. This phenomenon accounts for the high accuracy of binning of the high abundance genomes, which generally consisted of longer scaffolds that can be binned more accurately. This effect can also be observed in the simMC + dataset, in which only a small portion of scaffolds from low abundance genomes passed the MaxBin minimum length threshold, and therefore only the high abundance genomes were binned. Due to the poor assembly quality of simMC + (the N50 for simMC + is only 383 while the N50 for simLC + and simHC + are 1293 and 17169, respectively), only 11 bins were generated from the simMC + dataset compared to 57 and 78 bins from the simLC + and simHC + datasets.For simHC + dataset, which had an evenly distributed species abundance levels, MaxBin yielded 78 bins (Figure 3(C)) with 88.93% precision. In the simHC + dataset, there were fewer mis-assigned scaffolds than in simLC + (22.24 Mbps in simHC+; 53.24 Mbps in simLC+). The appearance of unclassified sequences in some bins, which was measured at 62.5 Mbps, was probably due to the similarity of abundance levels between different species, which resulted in a lowered sensitivity of 73.46%.

### Binning of datasets from the Human Microbiome Project

The Human Microbiome Project (HMP) was designed to document the microbial populations that occupy habitats in or on the human body. A total of 749 samples were generated for selected corporeal habitats, including the gut, the mouth, the vagina, and the skin [39]. MaxBin was applied to identify genomic bins from three selected HMP datasets with very different sample sizes: the tongue dorsum sample SRS013705, the subgingival plaque sample SRS014477, and the stool sample SRS018656. The reads and assembled sequences were downloaded from HMPDACC website (http://hmpdacc.org/). These three samples were chosen to compare the performance of MaxBin on real metagenomic datasets with very different amounts of raw sequence reads (12 GB for SRS013705, 1.4 GB for SRS014477, and 6.4 GB for SRS018656). MaxBin yielded 31 bins for SRS013705, 4 bins for SRS014477, and 10 bins for SRS018656, consistent with the total base pairs of scaffolds that passed the minimum length threshold (83 MB for SRS013705, 14 MB for SRS014477, and 45 MB for SRS018656). The number of unclassified sequences for SRS013705, SRS014477, and SRS018656 was 11.18, 0.37, and 2.17 Mbps. The taxonomy of the bins was analyzed using MEGAN4 [40] and compared to the results of the HMP community profiles (also downloaded from HMPDACC website) generated by fragment recruitment of the metagenomic sequencing data to related sequenced isolates [41]. The binning results and the closest species are listed in Additional file 1: Table S3. In general, MaxBin successfully generated bins for the high abundance populations in each sample. For instance, three of the four most abundant predicted species in SRS013705 were successfully binned, including populations related to *Prevotella melaninogenica*, *Streptococcus salivarius*, and *Veillonella dispar*. Similarly, in SRS014477, species among the four recovered genomes included *Treponema denticola*, *Treponema vincentii*, *Corynebacterium matruchotii*, and *Bacteroidetes* oral taxon 274, which were identified as the most abundant species in this sample. In the SRS018656 dataset, three of the four most abundant species were isolated as well, including *Dialister invisus*, *Bacteroides cellulosilyticus*, and *Ruminococcus* sp., [see Additional file 1: Table S3]. The MaxBin threshold on the minimum scaffold lengths limited the number of isolated bins that could be extracted from these datasets, including several strains of *Rothua dentocariosa* in SRS014477 and *Fusobacterium* sp. in SRS013705. Nevertheless MaxBin was still capable of isolating most of the high abundance genomes from real metagenomic samples with variable sequencing depths and different species composition complexity.

We compared our binning results to an approach using emergent self-organizing maps (ESOM), [2,8,9]. An ESOM graph was generated for the tongue dorsum sample SRS013705 based on tetranucleotide frequencies [2], and the sequences on the graph were colored based on the MaxBin binning results [see Additional file 1: Figure S5]. Since ESOM relied on the contour boundaries to distinguish binned genomes, we focused on observing whether the bins generated by MaxBin were consistent with the original ESOM boundaries. Indeed, comparison between ESOM [see Additional file 1: Figure S5(A)] and MaxBin binning results [see Additional file 1: Figure S5(B)] demonstrated that most MaxBin bins (shown as different colors) overlaid with the ESOM boundaries, confirming the ability of MaxBin to separate genomes according to their genomic signatures. In addition we also observed that closely related populations, such as the three bins representing three *Prevotella* species and two bins belonging to the *Leptotrichia* species, were not successfully resolved using the ESOM approach, suggesting that MaxBin could further distinguish genomes with similar tetranucleotide frequencies based on scaffold coverage levels and single-copy marker genes.

### Recovering genomes from cellulolytic consortia using MaxBin

MaxBin was also applied to metagenomes obtained from replicates of enriched cellulolytic consortia derived from green waste compost. These enriched communities have yielded deeply sampled metagenomic datasets from which individual genomes can be recovered [30,42,43]. Enrichments were performed at 37°C degrees over multiple passages and DNA was isolated from two replicates, termed samples 37A and 37B, of the second passage for metagenomic sequencing. Metagenomes obtained from both 37A and 37B achieved reasonable assembly quality - the N50 for the two replicates were 2,907 and 1,994 bps, respectively. MaxBin was then used to bin the assembled metagenomics data from the two replicates. The most abundant 10 genomes of both replicates are shown in Table 2. The complete list of bins and their genomic properties can be found in Additional file 1: Table S4. The amount of unclassified sequences of 37A and 37B were measured at 2.45 Mbps and 4.36 Mbps, respectively, excluding scaffolds that were shorter than 1,000 bps.

**Table 2:** Most abundant species in sample 37A and 37B

**37A**				**37B**			
**Bin number**	**Species**	**%**^**a**^	**Completeness**	**Bin number**	**Species**	**%**^**a**^	**Completeness**
001	*Sorangium* sp.	48.1%	95.30%	001	*Niastella* sp.	26.6%	94.40%
002	*Niastella* sp.	11.4%	95.30%	002	*Teredinibacter* sp.	12.7%	99.10%
003	*Opitutus* sp.	7.5%	84.10%	003	*Sphingomonas* sp.	10.0%	96.30%
004	*Chitinophaga* sp.	6.5%	98.10%	004	*Cellulomonas* sp.	7.9%	83.20%
005	*Rhodanobacter* sp.	5.5%	88.80%	005	*Cellulomonas* sp.	7.8%	92.50%
006	*Cytophaga* sp.	4.3%	98.10%	006	*Chitinophaga* sp.	4.8%	98.10%
007	*Opitutus* sp.	2.7%	60.70%	007	*Rhodanobacter* sp.	4.6%	46.70%
008	*Oceanibaculum* sp.	2.6%	86.00%	008	*Pseudoxanthomonas* sp.	4.5%	95.30%
009	*Pelagibacterium* sp.	1.6%	66.40%	009	*Opitutus* sp.	3.0%	92.50%
010	*Cellulomonas* sp.	1.3%	89.70%	010	*Sorangium* sp.	2.5%	93.50%

^a^Relative abundance ratios; defined as actual coverage levels divided by summed coverage levels of all genomes.

**Figure 4:**
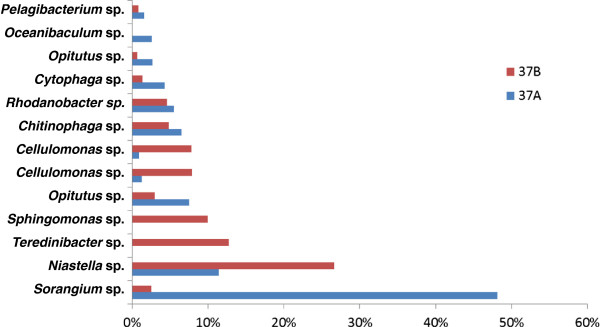
**Species distribution comparison between enriched cellulolytic compost replicates 37A and 37B.** Blue bars and red bars indicate that the species was found in 37A and 37B, respectively.

We found that even though the two replicates were enriched from the same compost inoculum, the species composition and the relative abundance ratios of these species diverged (Figure 4). In the 37A dataset, the most abundant bin was classified as *Sorangium* sp. followed by bins classified as *Niastella* sp. and *Opitutus* sp. In 37B, the most abundant species was classified as *Niastella* sp., which was a nearly identical bin to the one found in 37A, followed by *Teredinibacter* sp. and *Sphingomonas* sp. The *Sorangium* sp. bin, which occupied nearly half of the population in 37A (48.1%), was only found at 2.5% abundance in 37B. Furthermore, the second and third most abundant bins in 37B (*Teredinibacter* sp. and *Sphingomonas* sp.) were not observed in 37A. Note that the second most abundant species in 37B, *Teredinibacter* sp., is distantly related to *Teredinibacter turnerae* (with amino acid identity at 67.4%), an endosymbiotic cellulolytic gammaproteobacteria isolated from the gill tissue of a shipworm, *Lyrodus pedicellatus*[44].

The binning results obtained for MaxBin were compared with two other binning methods: ESOM (tetranucleotide frequencies) and differential coverage binning [7]. The ESOM graphs demonstrated that the MaxBin binning results fit extremely well with the ESOM boundaries for both replicates [see Additional file 1: Figure S6]. Differential coverage binning was performed on the *Sorangium* sp., *Niastella* sp. and *Opitutus* sp. bins since initial inspection of the metagenomic datasets revealed that the assembled sequences for these populations were nearly identical in 37A and 37B.

We inspected the *Sorangium* sp. genome that was recovered using MaxBin, ESOM graph, and differential coverage binning [see Additional file 1: Figure S7; Table 3]. The GC content of the three *Sorangium* sp. genomes, recovered by the three different approaches, were all 64%, consistent with the high G + C proportion observed for the genome of *Sorangium cellulosum* (71.4%) and other myxobacteria [45]. The genome sequences that MaxBin and ESOM recovered were more complete as compared to the differential coverage binning approach: the numbers of unique marker genes for MaxBin, ESOM, and differential coverage binning were 102, 102, and 99 out of all 107 marker genes. When we increased the minimum length threshold from 1,000 bps to 5,000 bps, the number of marker genes for MaxBin and differential coverage binning did not change (Table 3). We also extracted the *Niastella* sp. and *Opitutus* sp. genomes using differential coverage binning method and compared their genomic features in Additional file 1: Table S5.

**Table 3:** **Genome statistics of isolated *Sorangium *** **sp. using different binning methods**

	**Differential coverage (5,000 cutoff)**	**Differential coverage (1,000 cutoff)**	**MaxBin (1,000 cutoff)**	**MaxBin (5,000 cutoff)**	**ESOM (2,000 cutoff)**
Total length	4,500,845	4,588,758	5,001,615	4,893,814	5,122,074
Scaffold count	82	93	150	104	143
Mean length (bps)	54,888.4	49,341.5	33,568.84	47,514.0	36,070.9
Maximum length (bps)	240,486	240,486	240,486	240,486	240,486
% GC	63.9	63.8	64	64	63.8
Total marker gene count	103	104	109	107	107
Unique marker gene count	99	99	102	102	102

The sequences that were found only in MaxBin-derived bins compared to the differential coverage binning method were collected and analyzed by MEGAN to identify the taxonomy of these sequences. MEGAN analysis of the scaffolds not classified by differential coverage binning demonstrated that most of the scaffolds missed by the differential coverage binning approach were classified into the Proteobacteria lineage, as indicated in Additional file 1: Figure S8. Since the bins closest in abundance were affiliated with *Bacteroidetes* and *Verrucomicrobia*, the sequences affiliated with the Proteobacteria lineage shown in Additional file 1: Figure S8 likely belong to the *Sorangium* bin. Therefore, MaxBin collected a more complete *Sorangium* sp. genome compared to the differential coverage binning approach. We manually inspected the five scaffolds grouped into the *Bacteroidetes* lineage and found that the coverage levels of these scaffolds (974.7, 1990.5, 1019.9, 1027.4, and 1709.5) were much higher than predicted coverage of second abundant species, *Niastella* sp. (270.36). Since the coverage levels of these five scaffolds were much closer to that of *Sorangium* sp., their assignment may be consistent with belonging to *Sorangium* sp. bin. An expanded genome size obtained by MaxBin compared to differential binning was also observed for the *Niastella* and the *Opitutus* spp. genomes. In both cases, the majority of the additional sequences recovered by MaxBin were affiliated with the predicted lineage [see Additional file 1: Figure S8].

### *Sorangium* sp. bin represents the genome of an unusual myxobacterium

**Figure 5:**
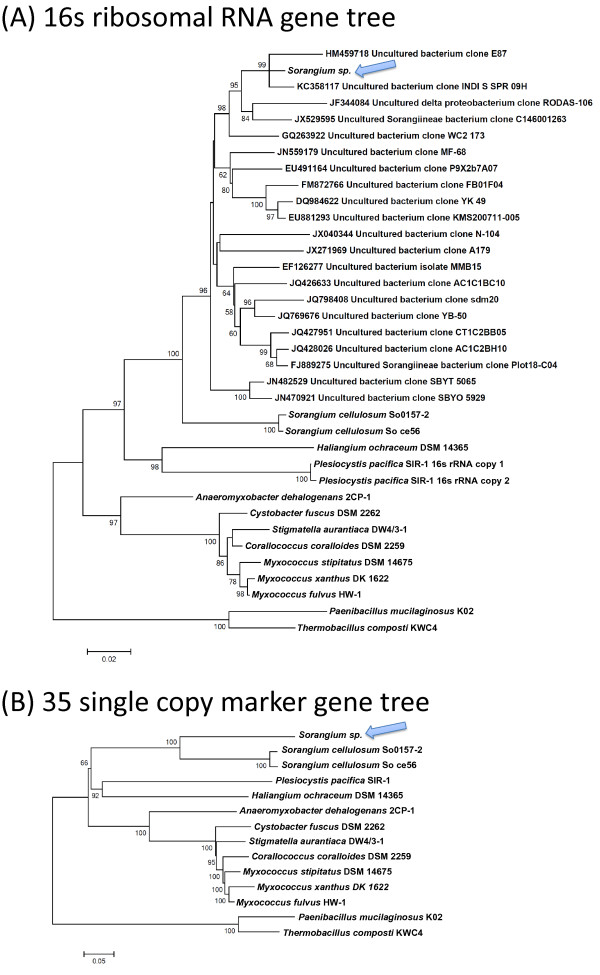
**Phylogenetic trees built for the species *Sorangium *sp. found in 37A and 37B.** Arrowheads indicate the whereabouts of *Sorangium* sp. in the trees. **(A)** 16S ribosomal RNA gene tree. **(B)** Concatenated gene tree for 35 protein-coding marker genes.

A complete 16S rRNA gene (90% identical to *S. cellulosum*) was recovered from the bin containing the *Sorangium sp.* genome and a phylogenetic tree was constructed to classify the bin (Figure 5(A)). Analysis of the phylogenetic tree demonstrated that the novel myxobacterial population was a Deltaproteobacterium in the *Myxococcales* order affiliated with the suborder *Sorangiineae,* but was distinct from the family *Polyangiaceae,* which contains the validated species *Sorangium cellulosum*, *Byssovorax cruenta and Chondromyces apiculatus*[46]. This new family in the *Sorangiineae* has no cultivated members and consists of 16S rRNA clones representing uncultivated species. The two 16S rRNA clones in this family that are most similar (99% identity) to that of *Sorangium* sp. were recovered separately from earthworm guts and large-discharge carbonate springs [Genbank: HM459718 and KC358117]. The phylogenetic classification of this bin was confirmed by construction of a concatenated gene tree from the genomic bin with 35 single-copy marker genes, which confirmed that it was distantly related to *Sorangium cellulosum* (Figure 5(B)). Surprisingly, the MaxBin binning results, supported by complementary binning by ESOM and differential coverage binning methods, demonstrated that the *Sorangium* sp. genome was approximately 5 MB, while the two sequenced strains of *Sorangium cellulosum* have genomes of 13.0 MB (strain So ce56) and 14.7 MB (strain So0157-2). Genomes of 11 myxobacterial genomes were compared, and 193 genes were identified as universally shared. For those 193 genes, 158 genes were found to be present in *Sorangium* sp., suggesting that despite its significantly smaller size, this genome still contains most of the common genes found in myxobacteria.

**Figure 6:**
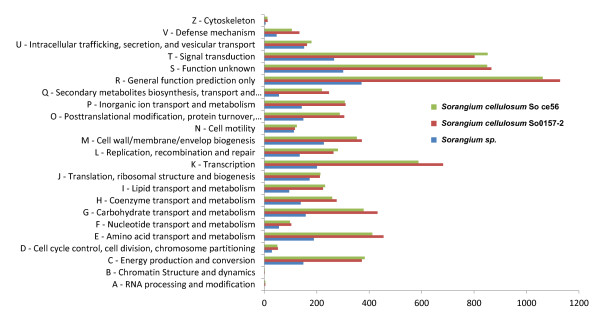
**Comparison of the numbers of clusters of orthologous groups (COG) families predicted from the genes extracted from the three *Sorangium *** **species.**

We compared the gene content between the three *Sorangium* species to further understand their differences. COG families of all extracted genes from the three *Sorangium* species were identified and compared. The identified COGs were classified into different functional categories for the three species, as depicted in Figure 6 (the actual numbers can be referred to in Additional file 1: Table S6). Since the genome sizes of the two *Sorangium cellulosum* genomes were about 2.5 times larger than the recovered *Sorangium* sp. genome, gene numbers for individual functional categories were expected to be approximately 2.5 times larger for *S. cellulosum* isolates. Indeed, we observed that the number of genes in COG categories for the two *Sorangium cellulosum* species were, on average, 2.35 times more than those in *Sorangium* sp. (7,535 and 7,255 genes in all COG categories for the two *Sorangium cellulosum* strains compared to 3,155 genes for *Sorangium* sp.). Among the COG categories, gene numbers differed most for COG group Q (Secondary metabolites biosynthesis, transport, and catabolism); the two *Sorangium cellulosum* isolate genomes averaged 4.17 times more assigned genes than the recovered *Sorangium* sp. genome. Production of large numbers of secondary metabolites is a characteristic of myxobacterial metabolism, and *Sorangium cellulosum* isolates in particular produce antifungal and antibacterial compounds [47]. The only other myxobacterial isolate with a similarly sized genome to the uncultivated *Sorangium* sp. genome is *Anaeromyxobacter dehalogens* (5.01 MB) [48]. *A. dehalogens* is characterized by an extensive genomic repertoire for anaerobic respiration, including growth with nitrate, halogenated organics and metals as terminal electron acceptors. The uncultivated *Sorangium* sp. genome lacked genes for denitrification, sulfate reduction, and metal reduction, indicating that it may primarily have an aerobic lifestyle [see Additional file 1: Figure S9].

**Figure 7:**
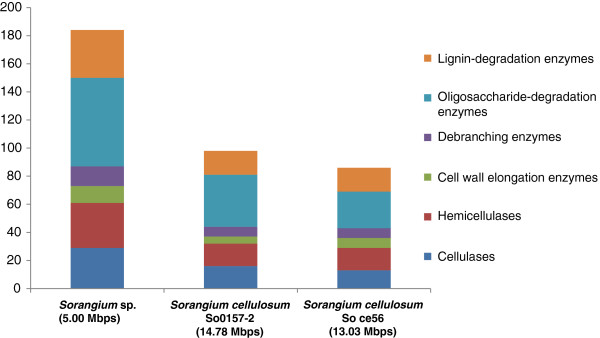
**The number of cellulose-, hemicellulose-, and lignin-degradation genes extracted from the three *Sorangium *species.** Genome sizes are indicated below the species names.

In contrast to the general survey provided by COG family comparisons, extraction of glycoside hydrolase (GH) and auxiliary activity (AA) enzymes using the CAZy database demonstrated that the number of genes relevant to biomass deconstruction was, on average, doubled in the uncultivated *Sorangium* sp (Figure 7). In particular, genes that cluster with glycoside hydrolase families specifically involved in cellulose hydrolysis (GH5, 6, 7, 9, 44, 45, 48) were twice as abundant (29 versus 13 and 16) in the uncultivated *Sorangium* sp. genome compared to the *S. cellulosum* isolate genomes (the actual number of all GH genes are listed in Additional file 1: Table S7).

## Discussion

MaxBin provides an automated method to recover genomes from individually assembled metagenomic datasets by combining information from tetranucleotide frequencies and scaffold coverage levels, which were used in previous binning methods, including the ESOM and differential binning approaches [7]. Most of these previous binning methods were based on visualization of scaffolds, and the bins were selected manually from the identified boundaries or clusters of scaffolds. Several recent studies based on Stochastic Neighbor Embedding (SNE) achieved a better clustering of metagenomic scaffolds and were partially automated by human-automated polygon selection method [49,50]. To the best of our knowledge, MaxBin is the only fully automated metagenomic scaffold binning software package that only requires users to input assembled scaffolds and the coverage levels of these scaffolds. MaxBin can also calculate the coverage levels of scaffolds automatically if sequencing reads are provided. Even though some other automated metagenomic binning tools exist, such as AbundanceBin [10] or MetaCluster [11,12], they are all designed to classify sequencing reads, not assembled scaffolds.

The tetranucleotide frequency is a genomic signature that is most commonly used for binning purpose. In principle MaxBin can accommodate different genomic signatures, including hexanucleotide signatures, which have been used in other binning algorithms (for example, CompostBin [51]). However, ESOM binning studies have demonstrated that binning by tetranucleotide frequencies best balances phylogenetic resolution with ease of computational processing [2,52]. We therefore decided to use tetranucleotide frequencies for binning purpose in the MaxBin algorithm.

Due to the high dimensionality of the feature space and the associated problems of dimensionality in the use of tetranucleotide frequencies, shorter fragments will produce noisy frequency profiles and have resulted in poor performance [51]. Therefore, most binning methods based on tetranucleotide frequencies will filter out short sequences based on different minimum length cutoff settings. For example, the minimum length cutoff threshold of ESOMs is 3,000 bps while thresholds as low as 500 bps were used for time series ESOMs, which combined eleven metagenomic datasets [9]. For differential coverage binning approach, thresholds of 2,000 to 5,000 bps were applied. The thresholds are necessary in all these binning methods to prevent incorrect binning of short scaffolds; however, application of a threshold that is too stringent will discard relevant sequences to recover more complete genomes. Since our tests on applying different length cutoff values on all simulated datasets revealed that 1,000 bps threshold yielded the highest sensitivity value [see Additional file 1: Figure S3], the threshold for scaffold length for MaxBin was determined to be 1,000 bps. For MaxBin, binning may be performed at different thresholds and the results compared, as was demonstrated for the *Sorangium* sp. bin, to determine the proper threshold to achieve correct and comprehensive binning.

To find the metric for comparing whether two sequences were sampled from the same genome or from different genomes, we performed an experiment to find the distributions of tetranucleotide frequency distances for both intra- and inter-genome distances. We then identified the mean and standard deviation values and inserted them into the probability function of the expectation-maximization algorithm. The Shapiro-Wilk test rejected the null hypothesis that the intra- and inter-genome distances were normally distributed (W = 0.81 and 0.93). However, the differences between intra- and inter-genome distributions, as shown in Additional file 1: Figures S1(A) and S1(B), are large enough to distinguish intra- and inter-genome tetranucleotide frequency distances. Therefore, the mean and standard deviation values were inserted into equations for the Gaussian distributions for both intra- and inter-genome distances in order to estimate the probability that any two sequences were sampled form the same genome. This procedure was also supported by analysis of our binning performance on the simulated and real metagenomic datasets. We will continue to look for the most suitable probability function for further refinement of the MaxBin algorithm.

MaxBin also incorporates scaffold coverage levels as well as tetranucleotide frequencies. The scaffold coverage levels have been incorporated in the binning procedures for recovering genomes from a number of metagenomic datasets [7,9,42]. An important advantage of scaffold coverage levels is that it distinguishes sequence fragments extracted from species with similar tetranucleotide frequencies. For example, in Additional file 1 Figure S5(B) we showed that at least three *Prevotella* species and two *Leptotrichia* species were grouped together on the ESOM map, based on tetranucleotide frequencies, but were separated by MaxBin. MaxBin was able to separate these bins with similar tetranucleotide frequency profiles using scaffold coverage levels, which reflected the genomic coverage levels in sequences recovered from the human microbiome samples. We note that using the scaffold coverage levels for binning has one caveat: shared genomic regions between different genomes may have elevated coverage levels and confused the binning. The situation will be exacerbated if there are strains of the same species in the metagenome datasets, which will generate scaffolds with combined coverage levels. Theoretically, strains from the same species may still be binned separately using MaxBin if the assembly algorithms can recognize sequencing reads that belong to each strain; however, widely used assembly tools still cannot identify sequences from different strains and cannot assemble them separately. We attempted to assemble a simulated dataset with two different strains from the same species with very different coverage settings along with eight other species using Velvet (K = 55); however Velvet assembled most of the sequences from the two strains together and created scaffolds with much higher coverage levels than the actual coverage levels of the two strains. With the development of more advanced assembly tools, MaxBin will be capable of generating separate strain-level bins based on differences in coverage. We note that genome-amplified or *in vitro* normalized samples will not be binned accurately by MaxBin since the amplification and normalization process may disrupt the actual scaffold coverage levels and confuse both the assembly algorithms and MaxBin.

To find the number of bins in a metagenome, which is one of the most crucial parameters in MaxBin, we predicted genes from the metagenomes, identified 107 single-copy marker genes using HMMER3, estimated the number of marker genes in the metagenome, and took the median value among all marker gene counts as the number of bins in the metagenomes. We note that this method is based on heuristics, and the parameter is greatly influenced by the assembly quality, in which low quality assemblies will result in lower marker gene counts. Furthermore, there are also other single-copy marker gene sets, such as 35 marker genes used in predicting effective genome size in metagenomic samples [35] and 40 marker genes identified in [53]. Even though our median-number-based heuristic method and the 107 marker genes work well in MaxBin, we will keep exploring other more robust methods or more suitable marker gene sets for identifying the number of bins in metagenomes in our future works.

The binning performance of MaxBin also depends heavily on assembly quality. For example, MaxBin only generated 11 bins from the simulated simMC + dataset since this dataset was poorly assembled compared to simLC + and simHC+. High quality assemblies yield longer scaffolds, which will pass the minimum length threshold for MaxBin and have less-biased tetranucleotide frequencies. Recent developments in assembling metagenomic sequences [54-56] and uneven coverage single cell sequences [57] should improve the binning performance of MaxBin and other binning algorithms. Recent work has reported that preassembly filtering approaches, including digital normalization and read partitioning, produce higher assembly quality in two large soil metagenomes [58]. These advances suggest that MaxBin and other binning algorithms will be able to recover genomes from highly complex natural samples.

MaxBin was capable of binning high abundance populations obtained from metagenomic datasets from three distinct samples obtained in the Human Microbiome Project. These bins were consistent with previous genome assignments based on fragment recruitment to isolate genomes and validate the ability of MaxBin to recover individual genomes [41]. The inability to obtain bins for lower abundance populations from the HMP metagenomes was also seen in the simulated dataset 20X and simMC+, in which MaxBin was unable to bin genomes with lower abundance levels, which tend to have fewer scaffolds that pass the minimum length threshold for assignment by MaxBin.

Binning population genomes that represent 54% of the assembled sequence was achieved with the replicates of consortia adapted to grow on cellulose as a sole carbon source. These simple bacterial communities provided an opportunity to compare comprehensively the performance of MaxBin to the ESOM (tetranucleotide) and differential coverage approaches. Mapping the MaxBin-assigned bins onto the ESOM results demonstrated that MaxBin was capable of reproducing the individual bins denoted by the map contours and was able to distinguish closely related bins (for example, two *Cellulomonas* sp. in the 37A dataset) not resolved by the ESOM method [see Additional file 1: Figure S6(B)]. A detailed comparison of the ESOM-derived bin for the *Sorangium* sp. with the MaxBin-derived bin in the 37A dataset revealed that the ESOM bin recruited sequences that had approximately 100-fold coverage (the coverage of *Sorangium* sp. bin is estimated to be more than 1,000-fold), indicating that sequences with 100 or lower coverage levels were not binned correctly and accounted for the larger predicted genome size (5 versus 5.12 MB). In contrast, differential coverage binning recovered a smaller predicted genome (4.59 MB versus 5 MB); however, the additional scaffolds from the MaxBin results were shown to affiliate with the *Sorangium sp.* based on MEGAN analysis of the translated protein sequences in these scaffolds. MaxBin also recovered an expanded set of scaffolds compared to differential coverage binning for the *Niastella* sp. and *Opitutus* sp. bins present in both replicates. A key advantage of the MaxBin method compared to the differential coverage binning method for these datasets is that the *Terednibacter* sp. and *Sphingomonas* sp. bins were only present in the 37B sample, so these genomes could not be recovered using the differential coverage binning method.

A surprising result from the replicate cellulolytic consortia, common to all three binning methods, was the recovery of a myxobacterial genome distantly related to *Sorangium cellulosum* that was predicted to be approximately 5 MB. Most myxobacterial genomes are >9 MB [59] and the two most closely related genomes to this bin from strains of *Sorangium cellulosum* are >13 MB. The relatively large genome sizes of myxobacteria are consistent with their social activities and their ability to adapt to multiple environments [47]. The only myxobacterial genome of comparable size is *Anaeromyxobacter dehalogens*, which has a mosaic genome that combines specific genes of the myxobacteria with a versatile anaerobic metabolism typical of other Deltaproteobacteria [48]. Since *Sorangium* sp. does not possess genes required to live in an anaerobic environment, the reduction in genome size compared to other myxobacteria may arise from a different mechanism compared to *A. dehalogens*. The significantly reduced genome and the gene content of the recovered genome of the novel *Sorangium* sp. is consistent with a lifestyle that does not require the complex adaptations to respond to multiple environmental stimuli and may lack the social organization typically observed in myxobacteria. The observed dominance of the *Sorangium* sp. in one of the cellulolytic consortia and the expansion of genes for biomass deconstruction relative to sequenced *Sorangium cellulosum* strains suggests that this uncultivated population is specifically adapted to deconstruct plant biomass in natural environments than *Sorangium cellulosum*. Detailed comparative studies between the recovered genome of this unusual *Sorangium* sp. and other myxobacteria, as well as isolation of representatives of this uncultivated family, may expand our understanding of the evolution and the emergence of complex adaptive traits characteristic of the myxobacteria.

Currently, MaxBin is not able to bin viruses or plasmids, which lack the prokaryote marker genes to initiate the expectation-maximization algorithm. To alleviate this deficiency, we will refine MaxBin by adding genomic features that are distinct from these marker genes to bin viral genomes and plasmid sequences from metagenomic datasets in our future works. Also, the performance of MaxBin is greatly influenced by the estimation of the number of bins from the marker genes and the tetranucleotide frequency distance distributions. Improving the estimation of marker genes and identifying a more suitable distance distribution function will improve the algorithm and make it a generally applied tool for the recovery of individual genomes from metagenomic datasets.

## Conclusions

We have developed an automated binning algorithm that classifies assembled sequences in metagenomic datasets into recovered individual genomes. The algorithm was tested on several simulated and real metagenomes and shown to be highly accurate, comparing favorably to existing methods for metagenomic binning. Application to enriched cellulolytic consortia identified a number of uncultivated cellulolytic bacteria, including a myxobacterium that possessed a remarkably reduced genome and expanded set of genes for biomass deconstruction compared to its closest sequenced relatives. This work demonstrates that the processes required for recovering genomes from metagenomic datasets can be readily automated, an important advance in understanding the metabolic potential of microbes in natural environments.

## Availability of supporting data

The MaxBin program is available at https://sourceforge.net/projects/maxbin/. Metagenomic data for the enriched cellulolytic compost consortia are available at JGI IMG/M website (https://img.jgi.doe.gov/cgi-bin/m/main.cgi) under JGI taxon id 3300000869 (37A) and 3300001258 (37B). The MetaSim setting files, assembled scaffolds, and coverage files for replicating all simulation results, the binning results of HMP datasets that were mentioned in the text, and the binning results of the enriched cellulolytic compost metagenomes can be downloaded from the MaxBin download page (http://downloads.jbei.org/data/MaxBin.html).

## Abbreviations

COG: clusters of orthologous groups; KO: KEGG Orthology; KEGG: Kyoto Encyclopedia of Genes and Genomes; EM: expectation-maximization algorithm; ESOM: emergent self-organizing maps; GH: glycoside hydrolases; AA: auxiliary activities; HMP: Human Microbiome Project.

## Competing interests

The authors declare that they have no competing interests.

## Authors’ contributions

YWW, BAS, and SWS conceived the study and wrote the manuscript. YWW developed and maintained the MaxBin software package and carried out the analysis. YHT performed the enrichment experiments to generate the cellulolytic microbial consortia. SGT directed assembly and annotation of the metagenomes. All authors have read and approved the final manuscript.

## Supplementary Material

Additional file 1 MaxBin Supplementary Materials. Click here for file
