# Supplementary material for: MaxBin: an automated binning method to recover individual genomes from metagenomes using an expectation-maximization algorithm
Source: Microbiome. 2014 Aug 1;2:26. doi: 10.1186/2049-2618-2-26 (PMC4129434; doi:10.1186/2049-2618-2-26)
Supplement: Additional file 1 — MaxBin Supplementary Materials. [file 2049-2618-2-26-S1.pdf]

MaxBin Supplementary Materials – Wu et al., 2014

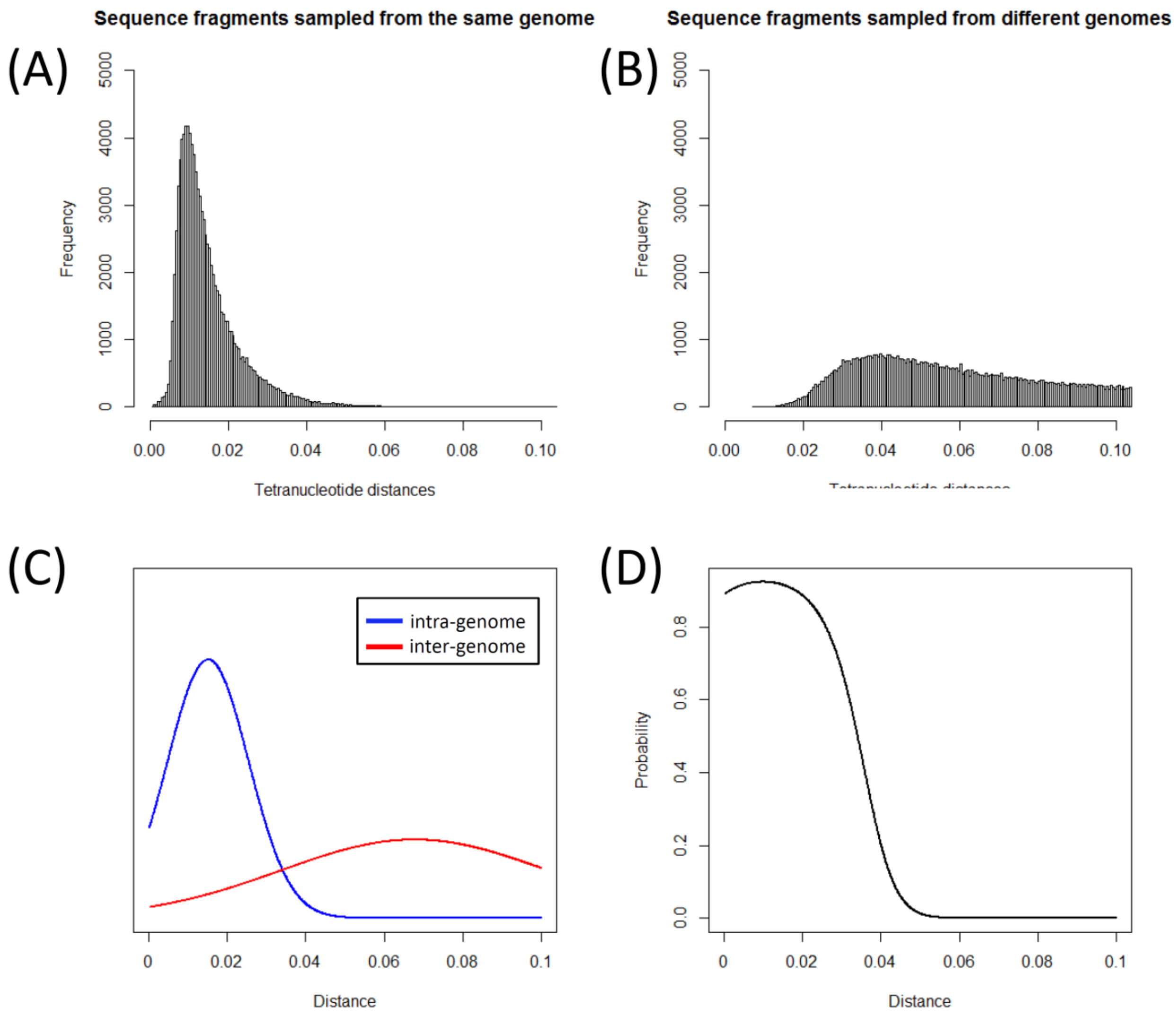

Figure S1. Euclidean distance distributions between sequences extracted from the same genome (intra-genome) and from two different genomes (inter-genome). (A) Histogram of distances between sequences sampled from the same genome. (B) Histogram of distances between sequences sampled from different genomes. (C) Approximate distance distributions of intra- and inter-genome sequences. (D) Probability distribution of two sequences belong to the same genome, which was obtained by calculating $\frac{P(distance \mid intra\_genome)}{P(distance \mid intra\_genome) + P(distance \mid inter\_genome)}$. See also Methods for details.

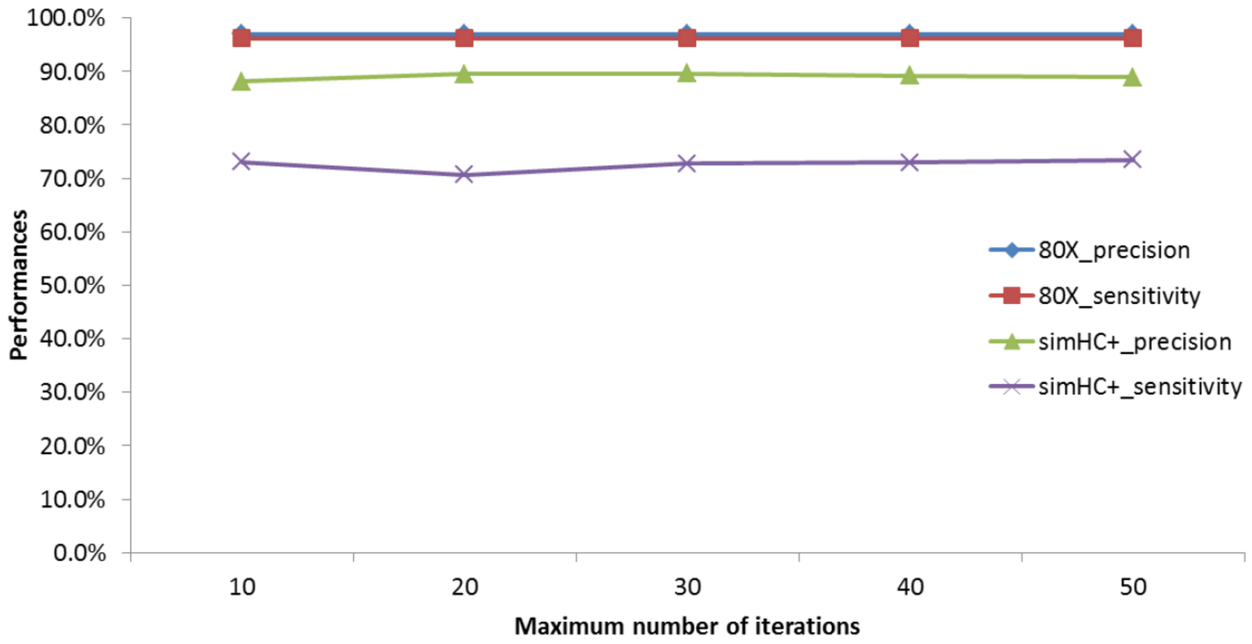

Figure S2. Binning performances (in terms of precision and sensitivity) for different settings of maximum number of iterations to run Expectation-Maximization algorithm.

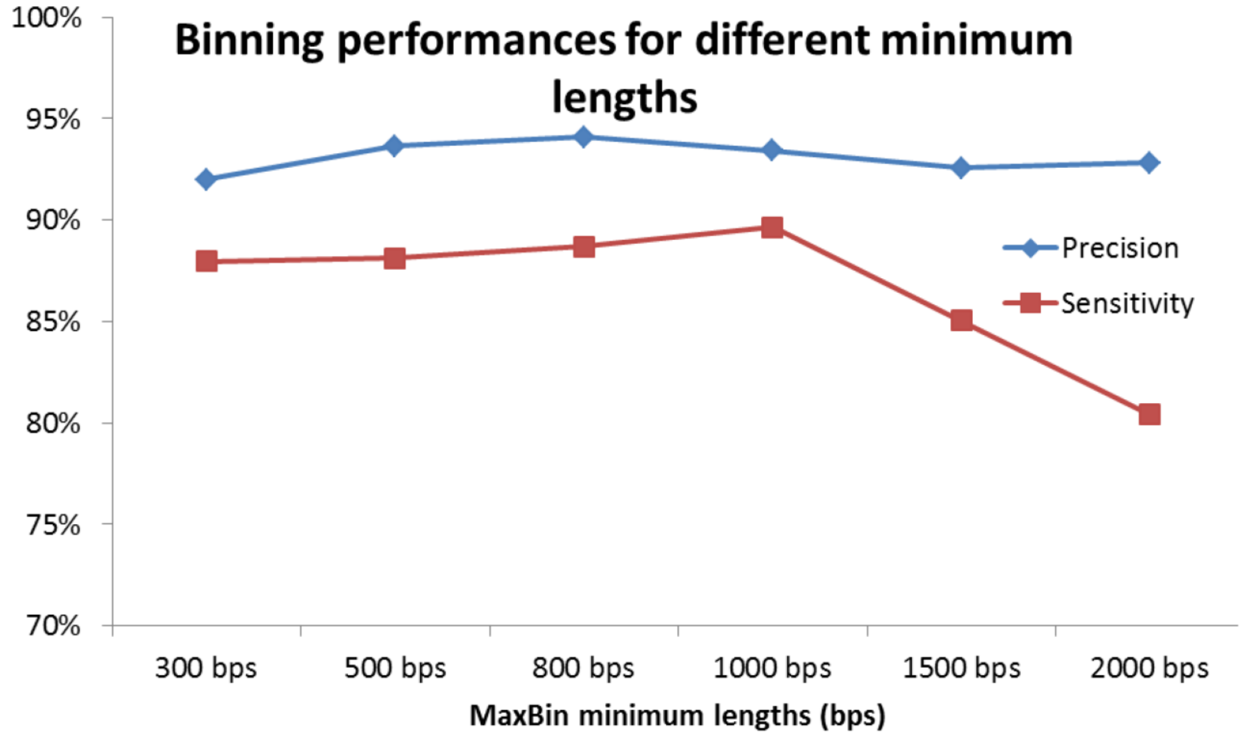

Figure S3. Precision and Sensitivity measured for different MaxBin minimum length cutoff values. The precision and sensitivity were estimated on all simulated datasets (including 80X, 20X, simLC+, simMC+, and simHC+) and were averaged to yield the values.

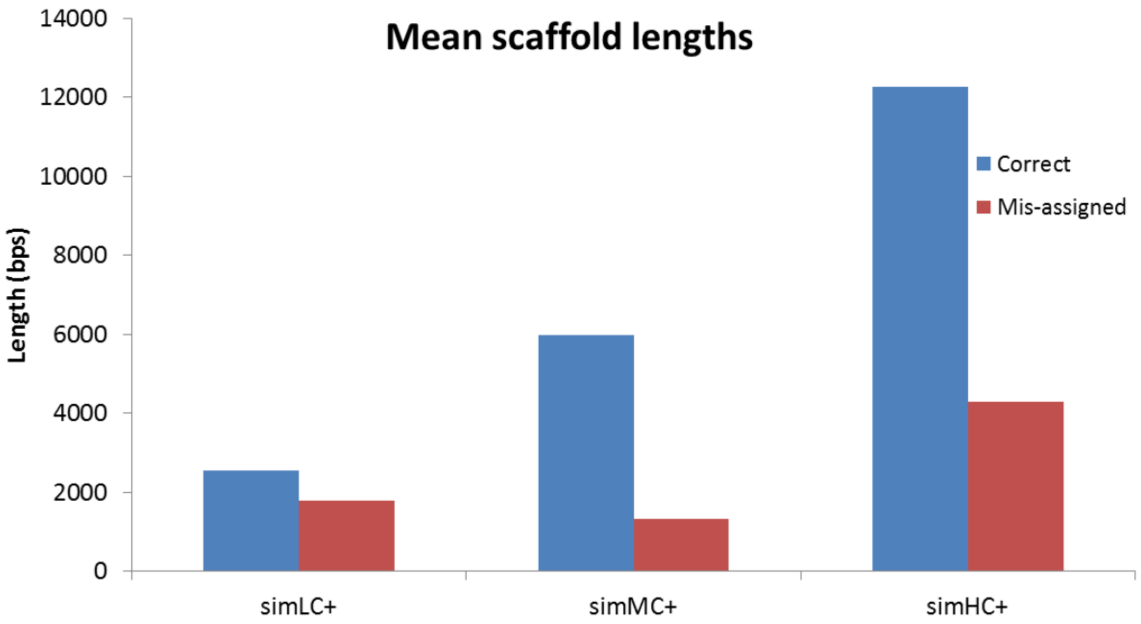

Dataset		Mean[1]	Std. dev. [1]	Max[1]	Min[1]
simLC+	Correct	2540.12	5662.25	245807	1000
	Mis-assigned	1785.92	1048.93	40810	1000
simMC+	Correct	5988.06	13587.51	180068	1000
	Mis-assigned	1332.06	732.65	39625	1000
simHC+	Correct	12275.15	16735.6	389523	1000
	Mis-assigned	4277.26	5716.08	99124	1000

[1] Length units are all base pairs (bps)

Figure S4. Correctly-classified and mis-assigned scaffolds for the 100-genome simulated datasets, simLC+, simMC+, and simHC+. The distribution of the sequence lengths, including mean, standard deviation, maximum and minimum length values are provided in the table.

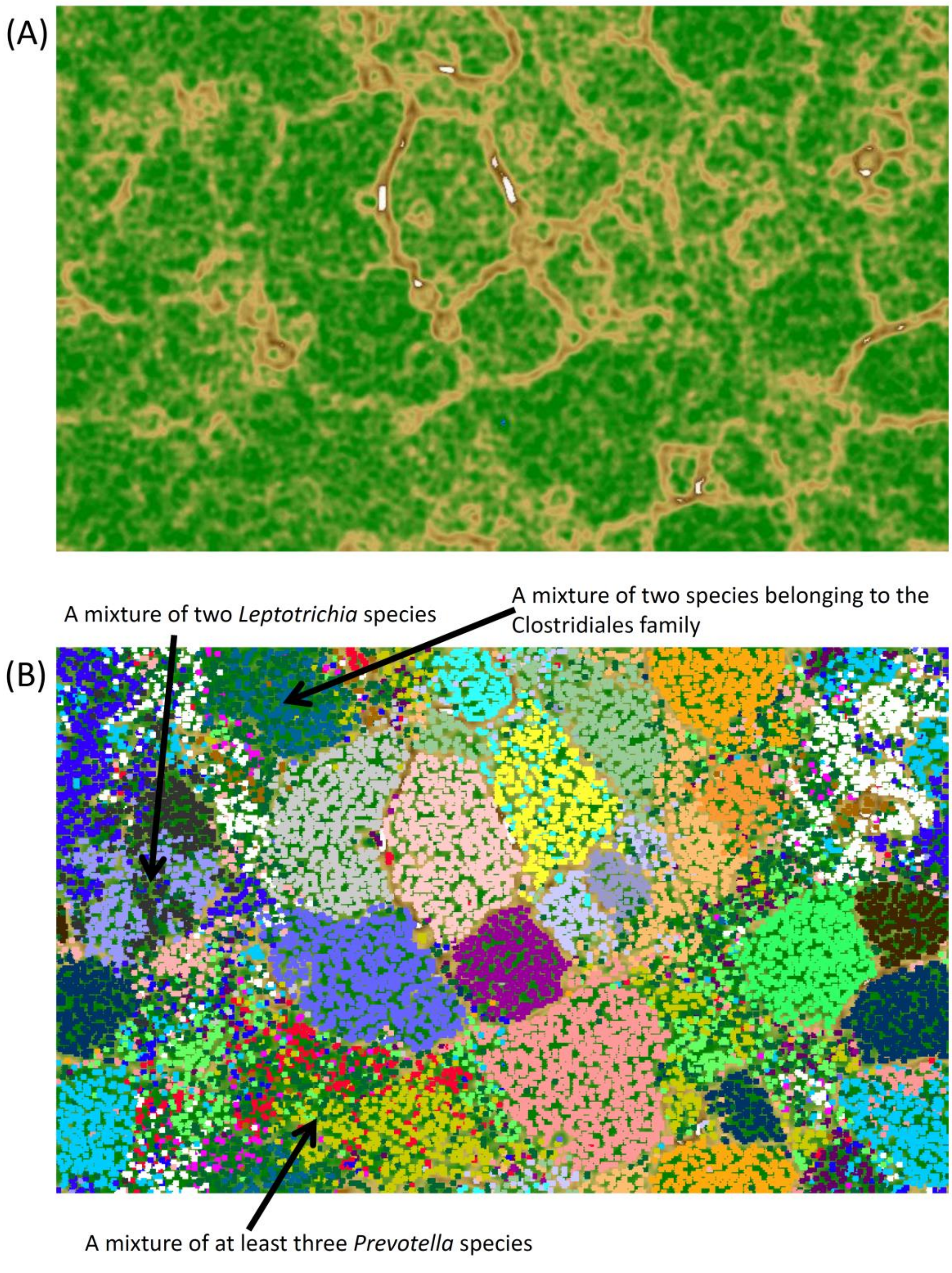

Figure S5. The emergent self-organizing maps (ESOM) binning results of the Human Microbiome Project (HMP) sample SRS013705. (A) Original ESOM plot. (B) Points colored based on MaxBin binning results. Different colors indicate different bins.

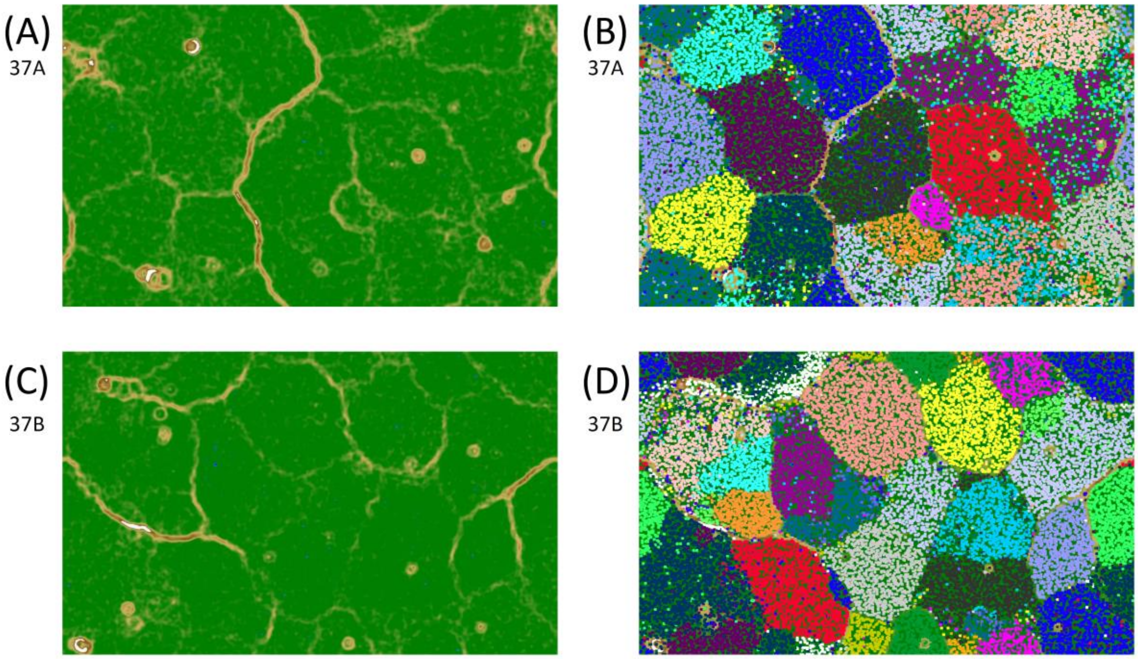

Figure S6. The emergent self-organizing maps (ESOM) binning results of 37A and 37B datasets. (A) Original ESOM plot of 37A. (B) 37A ESOM plot colored by MaxBin binning result. (C) Original ESOM plot of 37B. (D) 37B ESOM plot colored by MaxBin binning result.

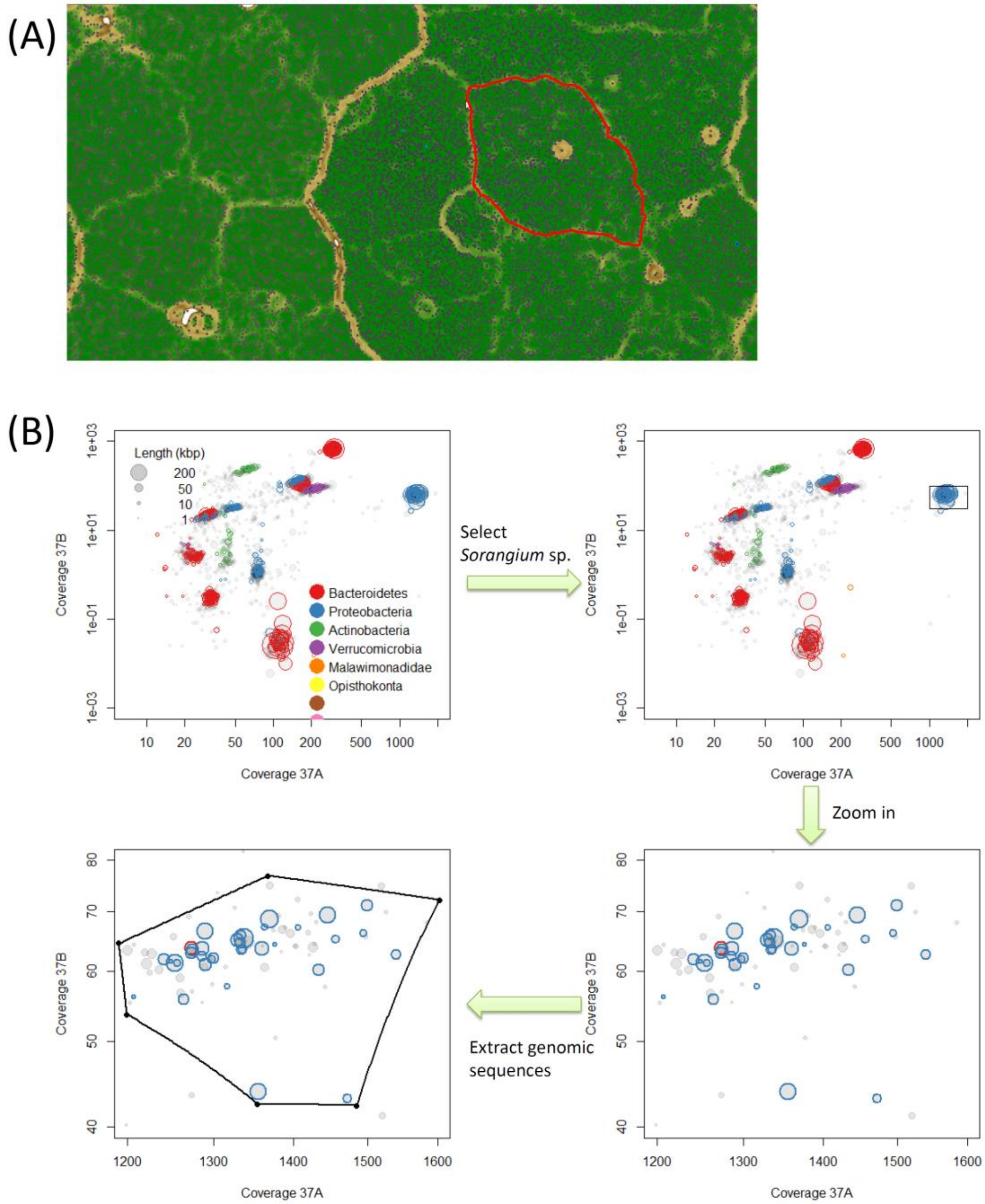

Figure S7. Steps of applying emergent self-organizing maps (ESOM) and differential coverage binning to extract *Sorangium* sp. genomic sequences. (A) Manually select the ESOM region belong to *Sorangium* sp. (B) Steps to draw differential coverage plot, select and zoom in to the region that belongs to *Sorangium* sp., and select the boundaries that include *Sorangium* sp. scaffolds.

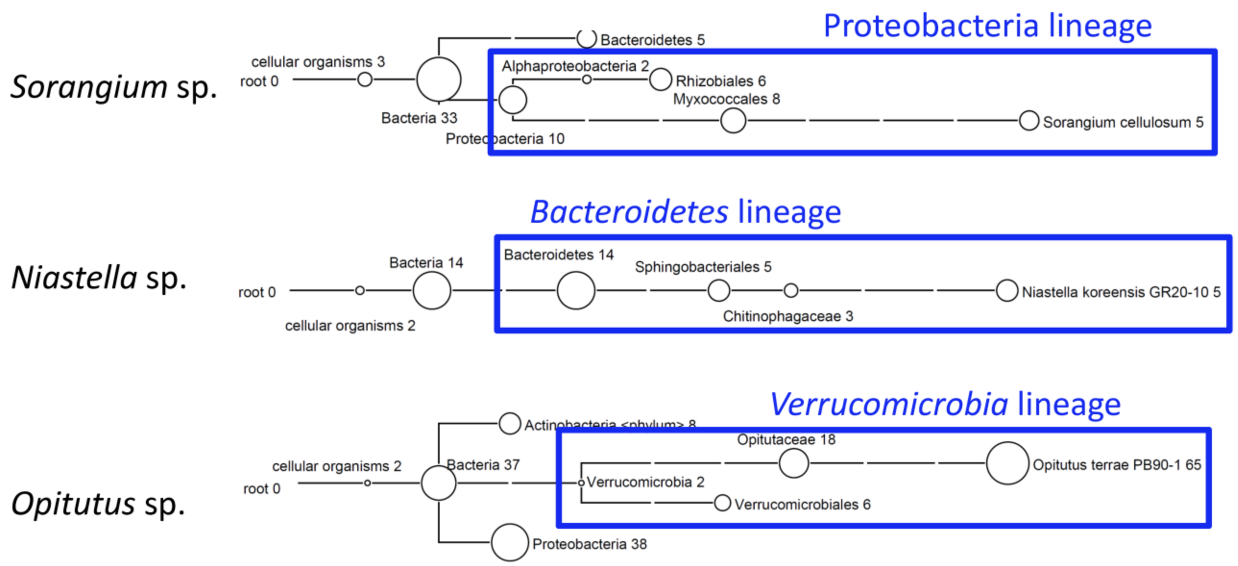

Figure S8. Using MEGAN to find the taxonomy of the scaffold sequences that were binned by MaxBin but missed by differential coverage binning approach. Numbers indicate the number of scaffolds that are assigned to corresponding taxonomy. Blue blocks indicate the lineages that most likely belong to the target species, which are *Sorangium* sp., *Niastella* sp., and *Opitutus* sp.

(A) Denitrification

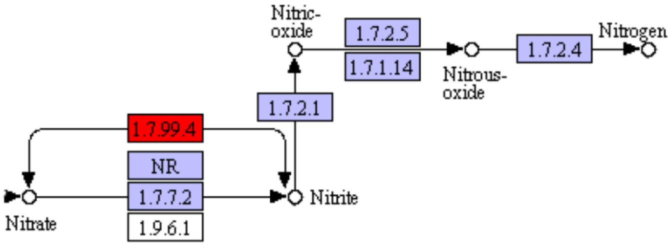

(B) Cytochrome C Oxidase

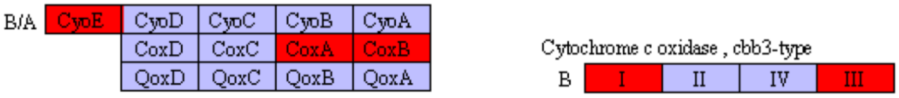

(C) Sulfate reduction

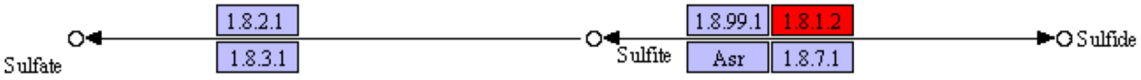

Figure S9. Pathway maps for denitrification, sulfate reduction, and cytochrome c oxidase in *Sorangium* sp. Red blocks indicate that the gene is present in the genome, and blue otherwise.

Table S1. Species and their relative abundance ratios used in 10-genome simulation

NCBI Genbank ID	Species name	Relative Abundance Ratios
256653503	*Acetobacter pasteurianus*	37.9%
347534971	*Flavobacterium branchiophilum*	18.9%
261854630	*Halothiobacillus neapolitanus*	15.2%
327384027	*Lactobacillus casei*	7.6%
300112745	*Nitrosococcus watsonii*	3.8%
325278757	*Odoribacter splanchnicus*	3.8%
111017022	*Rhodococcus jostii*	3.4%
182682970	*Streptococcus pneumoniae*	3.4%
307723218	*Thermoanaerobacter sp*	3.0%
154243958	*Xanthobacter autotrophicus*	3.0%

Table S2. Species and their relative abundance ratios used in 100-genome simulation.

NCBI Genbank ID	Species name	Relative Abundance Ratios		
		simLC+	simMC+	simHC+
256653503	*Acetobacter pasteurianus*	29.1%	14.5%	2.3%
330827700	*Aeromonas veronii*	6.2%	14.5%	1.9%
398314590	*Amycolatopsis mediterranei*	5.2%	11.6%	1.3%
308175814	*Arthrobacter arilaitensis*	2.5%	7.0%	1.2%
158421624	*Azorhizobium caulinodans*	1.4%	4.7%	1.2%
217957581	*Bacillus cereus*	1.1%	4.3%	1.1%
296500838	*Bacillus thuringiensis*	0.8%	1.2%	1.1%
42521650	*Bdellovibrio bacteriovorus*	0.8%	0.6%	1.1%
119025018	*Bifidobacterium adolescentis*	0.8%	0.6%	1.1%
295793053	*Bifidobacterium animalis*	0.7%	0.6%	1.1%
343385146	*Brachyspira intermedia*	0.7%	0.5%	1.1%
15791399	*Campylobacter jejuni*	0.7%	0.5%	1.1%
71082709	*Candidatus* Pelagibacter ubique	0.7%	0.5%	1.1%
194246403	*Candidatus* Phytoplasma mali	0.7%	0.5%	1.1%
256370581	*Candidatus* Sulcia muelleri	0.7%	0.5%	1.1%
297749010	*Chlamydia trachomatis*	0.7%	0.5%	1.1%
334694771	*Chlamydophila psittaci*	0.7%	0.5%	1.1%
325507407	*Clostridium acetobutylicum*	0.7%	0.5%	1.1%
331268188	*Clostridium botulinum*	0.7%	0.5%	1.1%
28209834	*Clostridium tetani*	0.7%	0.5%	1.1%

125972525	*Clostridium thermocellum*	0.7%	0.5%	1.1%
376247367	*Corynebacterium diphtheriae*	0.7%	0.5%	1.1%
385806437	*Corynebacterium pseudotuberculosis*	0.7%	0.5%	1.1%
334695745	*Corynebacterium ulcerans*	0.7%	0.5%	1.1%
284928601	*Cyanobacterium UCYN*	0.7%	0.5%	1.1%
307149945	*Cyanothece sp*	0.7%	0.5%	1.1%
46562128	*Desulfovibrio vulgaris*	0.7%	0.5%	1.1%
58616727	*Ehrlichia ruminantium*	0.7%	0.5%	1.1%
378937014	*Enterococcus faecium*	0.7%	0.5%	1.1%
336065242	*Erysipelothrix rhusiopathiae*	0.7%	0.5%	1.0%
209917191	*Escherichia coli*	0.7%	0.5%	1.0%
385805051	*Fervidicoccus fontis*	0.7%	0.5%	1.0%
302325342	*Fibrobacter succinogenes*	0.7%	0.5%	1.0%
347534971	*Flavobacterium branchiophilum*	0.7%	0.5%	1.0%
118496615	*Francisella novicida*	0.7%	0.5%	1.0%
156501369	*Francisella tularensis*	0.6%	0.5%	1.0%
19703352	*Fusobacterium nucleatum*	0.6%	0.5%	1.0%
333392846	*Gardnerella vaginalis*	0.6%	0.5%	1.0%
322433659	*Granulicella tundricola*	0.6%	0.5%	1.0%
148826757	*Haemophilus influenzae*	0.6%	0.5%	1.0%
301154649	*Haemophilus parainfluenzae*	0.6%	0.5%	1.0%
170717206	*Haemophilus somnus*	0.6%	0.5%	1.0%
12057215	*Halobacterium sp*	0.6%	0.5%	1.0%
261854630	*Halothiobacillus neapolitanus*	0.6%	0.5%	1.0%

261838873	*Helicobacter pylori*	0.6%	0.5%	1.0%
338736863	*Hyphomicrobium sp*	0.6%	0.5%	1.0%
385808586	*Ignavibacterium album*	0.6%	0.5%	1.0%
375256816	*Klebsiella oxytoca*	0.6%	0.5%	1.0%
332290650	*Krokinobacter sp*	0.6%	0.5%	1.0%
116332681	*Lactobacillus brevis*	0.6%	0.5%	1.0%
327384027	*Lactobacillus casei*	0.6%	0.5%	1.0%
104773257	*Lactobacillus delbrueckii*	0.6%	0.5%	1.0%
94986445	*Lawsonia intracellularis*	0.6%	0.5%	1.0%
296105497	*Legionella pneumophila*	0.6%	0.5%	1.0%
330833867	*Metallosphaera cuprina*	0.6%	0.5%	0.9%
124484829	*Methanocorpusculum labreanum*	0.6%	0.5%	0.9%
19918815	*Methanosarcina acetivorans*	0.6%	0.5%	0.9%
73667559	*Methanosarcina barkeri*	0.6%	0.5%	0.9%
239916571	*Micrococcus luteus*	0.6%	0.5%	0.9%
356592064	*Mycobacterium bovis*	0.6%	0.5%	0.9%
108796981	*Mycobacterium sp*	0.6%	0.5%	0.9%
400276727	*Mycoplasma gallisepticum*	0.6%	0.5%	0.9%
330723203	*Mycoplasma hyorhinis*	0.6%	0.5%	0.9%
308388224	*Neisseria meningitidis*	0.6%	0.5%	0.9%
300112745	*Nitrosococcus watsonii*	0.6%	0.5%	0.9%
325980881	*Nitrosomonas sp*	0.6%	0.5%	0.9%
54021964	*Nocardia farcinica*	0.6%	0.5%	0.9%
325278757	*Odoribacter splanchnicus*	0.6%	0.5%	0.9%

386720569	*Paenibacillus mucilaginosus*	0.6%	0.4%	0.9%
261403876	*Paenibacillus sp*	0.6%	0.4%	0.9%
54307237	*Photobacterium profundum*	0.5%	0.4%	0.9%
126695337	*Prochlorococcus marinus*	0.5%	0.4%	0.9%
347537839	*Pseudogulbenkiania sp*	0.5%	0.4%	0.9%
313496345	*Pseudomonas putida*	0.5%	0.4%	0.9%
116249766	*Rhizobium leguminosarum*	0.5%	0.4%	0.9%
111017022	*Rhodococcus jostii*	0.5%	0.4%	0.9%
380760311	*Rickettsia prowazekii*	0.5%	0.4%	0.9%
378722019	*Rickettsia rickettsii*	0.5%	0.4%	0.9%
374318767	*Rickettsia slovaca*	0.5%	0.4%	0.9%
99079841	*Ruegeria sp*	0.5%	0.4%	0.9%
194447306	*Salmonella enterica*	0.5%	0.4%	0.9%
269118642	*Sebaldella termitidis*	0.5%	0.4%	0.9%
114045513	*Shewanella sp*	0.5%	0.4%	0.9%
30061571	*Shigella flexneri*	0.5%	0.4%	0.9%
85057978	*Sodalis glossinidius*	0.4%	0.4%	0.9%
311222926	*Staphylococcus aureus*	0.4%	0.4%	0.9%
182682970	*Streptococcus pneumoniae*	0.4%	0.4%	0.9%
28894912	*Streptococcus pyogenes*	0.4%	0.4%	0.9%
354984442	*Streptococcus suis*	0.4%	0.4%	0.9%
116626972	*Streptococcus thermophilus*	0.4%	0.3%	0.9%
290954631	*Streptomyces scabiei*	0.4%	0.3%	0.9%
51891138	*Symbiobacterium thermophilum*	0.4%	0.3%	0.9%

320114857	*Thermoanaerobacter brockii*	0.4%	0.3%	0.9%
307723218	*Thermoanaerobacter sp*	0.4%	0.3%	0.9%
242397997	*Thermococcus sibiricus*	0.4%	0.3%	0.8%
239819985	*Variovorax paradoxus*	0.4%	0.3%	0.8%
323436265	*Weeksella virosa*	0.4%	0.3%	0.8%
225629872	*Wolbachia sp*	0.4%	0.3%	0.8%
154243958	*Xanthobacter autotrophicus*	0.4%	0.3%	0.7%
162418099	*Yersinia pestis*	0.3%	0.3%	0.4%

Table S3. Closest reference species of the binned genome from three Human Microbiom Project (HMP) samples

Bin	Closest reference species	Genome coverage	Completeness	Genome size	GC%
	SRS013705				
001	*Prevotella melaninogenica*	313.04	40.20%	1806112	40
002	*Streptococcus salivarius*	163.75	50.50%	1700440	36
003	*Neisseria flavescens*	123.35	52.30%	1583928	49
004	*Porphyromonas* sp. oral taxon 279	117.61	62.60%	1619277	60
005	*Actinomyces graevenitzii*	95.36	72.90%	1949837	58
006	*Streptococcus parasanguinis*	84.76	33.60%	2162969	41
007	*Veillonella atypica*	53.88	85.00%	3759355	36
008	*Leptotrichia buccalis*	49.1	68.20%	2550570	33
009	*Stomatobaculum longum*	46.03	67.30%	1773751	55
010	*Prevotella pallens*	42.53	89.70%	3702189	45
011	*Actinomyces* sp. ICM47	38.78	21.50%	2214274	65
012	*Leptotrichia hofstadii*	31.53	36.40%	2300441	29
013	*Treponema denticola*	31.37	98.10%	2148366	41

014	*Capnocytophaga* sp. CM59	26.3	86.90%	2727613	43
015	*Mogibacterium* sp. CM50	24.72	27.10%	819989	39
016	*Oribacterium* sp. ACB1	23.39	10.30%	1608463	43
017	*Johnsonella ignava*	22.28	69.20%	1882961	40
018	[*Eubacterium*] *infirmum*	21.37	67.30%	944042	42
019	*Gemella sanguinis*	21.32	61.70%	3475425	30
020	*Prevotella* sp. oral taxon 306	18.51	56.10%	3845362	45
021	*Lachnospiraceae* bacterium ICM7	17.17	31.80%	4836083	35
022	*Megasphaera micronuciformis*	16.16	65.40%	773971	46
023	*Capnocytophaga* sp. oral taxon 329	14.83	77.60%	3082884	41
024	*Prevotella salivae*	13.41	68.20%	3902144	41
025	*Lachnospiraceae* bacterium ICM7	12.91	57.00%	1955251	36
026	*Treponema vincentii*	12.86	92.50%	2614737	45
027	uncultured bacterium (gcode 4)	11.81	27.10%	1196134	36
028	*Peptostreptococcus stomatis*	10.99	32.70%	899206	39
029	*Prevotella* sp. oral taxon 473	10.78	65.40%	3294481	57
030	*Selenomonas sputigena*	10.75	45.80%	1189325	58

031	*Butyrivibrio proteoclasticus*	10.27	55.10%	3209210	49
	SRS014477				
001	*Bacteroidetes* oral taxon 274	15.33	86.90%	4365854	44
002	*Treponema denticola*	14.03	15.90%	1560979	32
003	*Corynebacterium matruchotii*	12.82	80.40%	3673816	55
004	*Treponema vincentii*	11.77	59.80%	4473939	46
	SRS018705				
001	*Dialister invisus*	198.47	59.80%	4521428	57
002	*Haloplasma contractile*	114.01	88.80%	1406648	29
003	*Ruminococcus bromii*	54.1	97.20%	2746565	41
004	*Subdoligranulum variabile*	48.21	77.60%	4978007	58
005	*Parabacteroides distasonis*	23.3	77.60%	3960717	46
006	*Bacteroides cellulosilyticus* (*fragilis*)	18.64	52.30%	9401347	46
007	*Bacteroides nordii*	16.11	40.20%	7278618	39
008	*Akkermansia muciniphila*	14.73	87.90%	2714789	57
009	*Alistipes shahii*	14.56	49.50%	4516450	60

010	*Clostridium leptum*	9.88	68.20%	1445238	48

Table S4. Complete list of species binned for 37A and 37B.

37A							
Bin#	Species	AA identity to closest species	Coverage	Relative abundance ratio (%)	Completeness	Genome size (bps)	GC content
001	*Sorangium* sp.	60.7% to *Sorangium cellulosum*	1144.24	48.1%	95.30%	5001615	64
002	*Niastella* sp.	73.1% to *Niastella koreensis*	270.36	11.4%	95.30%	4340643	48
003	*Opitutus* sp.	74.6% to *Opitutus terrae*	178.73	7.5%	84.10%	4971920	64
004	*Chitinophaga* sp.	64.9% to *Chitinophaga pinensis*	153.87	6.5%	98.10%	2915301	38
005	*Rhodanobacter* sp.	69.9% to *Rhodanobacter* sp. 115	130.42	5.5%	88.80%	3116221	69
006	*Cytophaga* sp.	62.9% to *Cytophaga hutchinsonii*	102.83	4.3%	98.10%	4813206	43
007	*Opitutus* sp.	74.5% to *Opitutus terrae*	63.95	2.7%	60.70%	2860184	65
008	*Oceanibaculum* sp.	65.6% to *Oceanibaculum indicum*	61.58	2.6%	86.00%	4800981	70
009	*Pelagibacterium* sp.	68.2% to *Pelagibacterium halotolerans*	38.24	1.6%	66.40%	3835464	66
010	*Cellulomonas* sp.	65.2% to *Cellulomonas fimi*	31.1	1.3%	89.70%	2906911	66

011	*Niastella* sp.	67.2% to *Niastella koreensis*	29.35	1.2%	92.50%	3269393	41
012	*Niastella* sp.	67.8% to *Niastella koreensis*	29.32	1.2%	94.40%	4579505	49
013	*Weeksella* sp.	66.2% to *Weeksella virosa*	23.89	1.0%	92.50%	3132025	39
014	*Parvibaculum* sp.	80.4% to *Parvibaculum lavamentivorans*	22.8	1.0%	80.40%	4114944	60
015	*Cellulomonas* sp.	73.6% to *Cellulomonas fimi*	21.75	0.9%	48.60%	1341252	72
016	*Mesorhizobium* sp.	75.7% to *Mesorhizobium alhagi*	21.51	0.9%	48.60%	2579242	68
017	*Opitutus* sp.	71.8% to *Opitutus terrae*	19.01	0.8%	38.30%	3499670	62
018	*Dyadobacter* sp.	67.5% to *Dyadobacter fermentans*	18.56	0.8%	76.60%	4364829	44
019	*Pseudoxanthomonas* sp.	87.4% to *Pseudoxanthomonas suwonensis*	18.32	0.8%	21.50%	1032550	70

37B

Bin#	Species	AA identity to closest species	Coverage	Relative abundance ratio	Completeness	Genome size (bps)	GC content
001	*Niastella* sp.	73.2% to *Niastella koreensis*	622.31	26.6%	94.40%	4338602	48
002	*Teredinibacter* sp.	67.4% to *Teredinibacter*	296.81	12.7%	99.10%	4268546	51

		turnerae					
003	*Sphingomonas* sp.	71.8% to *Sphingomonas wittichii*	233.2	10.0%	96.30%	2928274	68
004	*Cellulomonas* sp.	64.9% to *Cellulomonas fimi*	184.87	7.9%	83.20%	1907226	71
005	*Cellulomonas* sp.	65.8% to *Cellulomonas fimi*	181.24	7.8%	92.50%	2549936	66
006	*Chitinophaga* sp.	65.0 % to *Chitinophaga pinensis*	112.51	4.8%	98.10%	3590649	41
007	*Rhodanobacter* sp.	69.2% to *Rhodanobacter* sp. 115	107.18	4.6%	46.70%	2825683	69
008	*Pseudoxanthomonas* sp.	71.4% to *Pseudoxanthomonas suwonensis*	105.12	4.5%	95.30%	3591875	66
009	*Opitutus* sp.	74.6% to *Opitutus terrae*	69.14	3.0%	92.50%	5888819	64
010	*Sorangium* sp.	60.0% to *Sorangium cellulosum*	58.69	2.5%	93.50%	5113973	64
011	*Fluviicola* sp.	60.7% to *Fluviicola taffensis*	51.75	2.2%	98.10%	3781707	64
012	*Micavibrio* sp.	61.5% to *Micavibrio aeruginosavorus*	33.68	1.4%	99.10%	2453873	59
013	*Cytophaga* sp.	64.2% to *Cytophaga hutchinsonii*	33.17	1.4%	94.40%	5812044	39

014	*Opitutus* sp.	69.7% to *Opitutus terrae*	28.08	1.2%	93.50%	3702696	59
015	*Pelagibacterium* sp.	68.5% to *Pelagibacterium halotolerans*	26.42	1.1%	51.40%	2575205	66
016	*Cellulomonas* sp.	82.4% to [*Cellvibrio*] *gilvus*	23.09	1.0%	47.70%	2578944	74
017	*Niastella* sp.	67.5% to *Niastella koreensis*	21.06	0.9%	94.40%	3176136	41
018	*Rhodopseudomonas* sp.	77.7% to *Rhodopseudomonas palustris*	18.66	0.8%	57.00%	3646122	68
019	*Pelagibacterium* sp.	76.6% to *Pelagibacterium halotolerans*	18.61	0.8%	29.90%	484089	64
020	*Parvibaculum* sp.	81.2% to *Parvibaculum lavamentivorans*	16.67	0.7%	58.90%	1815727	60
021	*Sphingobacterium* sp.	71.1% to *Sphingobacterium* sp. 21	16.33	0.7%	22.40%	2941390	51
022	*Sphingobacterium* sp.	73.4% to *Sphingobacterium spiritivorum*	15.95	0.7%	62.60%	2980773	45
023	*Mesorhizobium* sp.	75.7% to *Mesorhizobium alhagi*	15.94	0.7%	53.30%	1305609	64
024	*Opitutus* sp.	69.6% to *Opitutus terrae*	15.51	0.7%	67.30%	2297225	68
025	*Phycisphaera* sp.	62.0% to *Phycisphaera*	15.29	0.7%	58.90%	3008188	59

		mikurensis					
026	*Pseudoxanthomonas* sp.	86.2% to *Pseudoxanthomonas suwonensis*	14.36	0.6%	15.00%	1305733	69

Table S5. Comparison between the binning results of *Niastella* sp. and *Opitutus* sp. produced by MaxBin and differential coverage binning approaches. The minimum length threshold is set to 1000 bps.

	Niastella sp.		*Opitutus* sp.	
	MaxBin	Diff. Cov.	MaxBin	Diff. Cov.
Total length	4340643	4174821	4972625	4249012
Scaffold count	134	91	488	281
Mean length (bps)	32639.3	45877.2	10210.7	15121
Maximum length (bps)	265826	265826	74880	74880
%GC	48	48	64	64
Total marker gene count	105	106	104	100
Unique marker gene count	102	105	90	99

Table S6. Numbers of genes for each clusters of orthologous groups (COG) category for the three *Sorangium* species.

	Sorangium sp.	S. *Cellulosum* So0157-2	S. *Cellulosum* So Ce 56
A - RNA processing and modification	1	4	4
B - Chromatin Structure and dynamics	0	2	2
C - Energy production and conversion	149	372	383
D - Cell cycle control, cell division, chromosome partitioning	29	52	50
E - Amino acid transport and metabolism	189	455	412
F - Nucleotide transport and metabolism	56	103	98
G - Carbohydrate transport and metabolism	158	432	379
H - Coenzyme transport and metabolism	139	276	259
I - Lipid transport and metabolism	95	224	232
J - Translation, ribosomal structure and biogenesis	173	212	214
K - Transcription	201	682	588
L - Replication, recombination and repair	135	264	281
M - Cell wall/membrane/envelop biogenesis	227	372	353
N - Cell motility	113	117	124
O - Posttranslational modification, protein turnover, chaperones	149	305	288
P - Inorganic ion transport and metabolism	143	310	307

Q - Secondary metabolites biosynthesis, transport and catabolism	56	247	220
R - General function prediction only	371	1128	1062
S - Function unknown	301	866	850
T - Signal transduction	266	802	852
U - Intracellular trafficking, secretion, and vesicular transport	152	163	180
V - Defense mechanism	47	134	105
Z - Cytoskeleton	5	13	12
Total	3155	7535	7255

Table S7. Numbers of extracted glycoside hydrolase (GH) and auxiliary activity (AA) genes in the three *Sorangium* species.

	Sorangium sp.	*Sorangium cellulosum* So0157	*Sorangium cellulosum* So Ce 56
GH1	12	7	5
GH2	3	2	1
GH3	10	6	4
GH5	21	11	10
GH6	1	1	
GH8	6	3	3
GH9	4	2	2
GH10	11	5	6
GH11	8	5	3
GH12	2	1	1
GH13	20	10	10
GH15	9	3	6
GH16	8	4	4
GH18	4	1	3
GH20	1	1	
GH23	11	6	5
GH25	1		1
GH26	3	1	2
GH28	3		3

GH30	4	2	2
GH31	6	4	2
GH32	3	2	1
GH33	6	2	4
GH35	2	1	1
GH39	2	1	1
GH42	2	1	1
GH43	31	18	13
GH44	3	2	1
GH52	1	1	
GH53	2	1	1
GH55	1	1	
GH57	2	1	1
GH62	7	4	3
GH63	2	1	1
GH65	2	1	1
GH67	2	1	1
GH73	1	1	
GH74	2		2
GH76	2	1	1
GH77	3	1	2
GH78	5	2	3
GH81	2	1	1
GH94	2	1	1

GH97	1	1	
GH105	2	1	1
GH109	13	7	6
GH113	3	2	1
GH114	3	1	2
GH115	3	2	1
GH116	1		1
GH117	1		1
GH120	1	1	
GH127	1	1	
GH128	1		1
GH131	1	1	
AA3	12	6	6
AA4	4	2	2
AA5	2	1	1
AA6	8	4	4
AA7	8	4	4

Table S8. Running time for all tested datasets.

Dataset	Total sizes of scaffolds (Mbps)	Assembly quality (N50)	Running time (hour:min:sec)[1]
10 genome simulation (80X)	39.06 Mbps	4574	00:11:58
10 genome simulation (20X)	18.51 Mbps	691	00:02:50
100 genome simulation (simLC+)	326.71 Mbps	1293	03:10:11
100 genome simulation (simMC+)	288.77 Mbps	383	00:19:17
100 genome simulation (simHC+)	319.26 Mbps	17169	02:39:15
HMP – SRS013705	157.46 Mbps	1078	01:03:22
HMP – SRS014477	39.47 Mbps	694	00:05:06
HMP – SRS018656	70.82 Mbps	1990	00:13:25
37A	109.52 Mbps	2907	00:33:25
37B	138.10 Mbps	1994	00:45:09

[1] The running time calculation assumes that the scaffold coverage information is readily available. It does not include the amount of time to map reads against scaffolds to obtain scaffold coverage information using Bowtie2.
